# Nested Evaluation of Sparse Multi-Objective Evolutionary Gene-Panel Discovery for Diabetic Nephropathy

**DOI:** 10.3390/bioengineering13101229

**Published:** 2026-10-21

**Authors:** Yilei Cong, Tengfei Wu, Xinyu Yang, Yiyun Zhu, Min Tang, Hua Yang, Ping Liu

**Affiliations:** 1 Department of Endocrinology, Longhua Hospital, Shanghai University of Traditional Chinese Medicine, Shanghai 200032, China; congyl123@126.com (Y.C.); lh02182@shutcm.edu.cn (T.W.); melodyyang1993@163.com (X.Y.); tangmin_longhua@163.com (M.T.); 2 Ward 2, Department of Nephrology, Longhua Hospital, Shanghai University of Traditional Chinese Medicine, Shanghai 200032, China; zyy9221@126.com; 3 Department of Cardiology, Longhua Hospital, Shanghai University of Traditional Chinese Medicine, Shanghai 200032, China

**Keywords:** diabetic nephropathy, gene expression profiling, feature selection, multi-objective optimization, nested cross-validation

## Abstract

**Background:** Diabetic nephropathy gene panels come from small kidney transcriptome cohorts, where screening and adaptive search inflate performance. Evolutionary multi-objective search balances panel size, accuracy, and biological coherence, but dense initialization slows it, and its benefit over ranking or regularized regression is rarely tested under nested evaluation. **Methods:** Six evolutionary algorithms optimizing leanness, inner balanced accuracy, and Gene Ontology (GO) coherence were evaluated with four repeats of nested five-fold cross-validation on GSE96804. Harmonic initialization was compared with Bernoulli, bounded uniform, and SparseEA initializers; GO was removed or randomized. Comparators were random, top-ranked differentially expressed gene (top-DEG), regularized, and wrapper panels and a permuted-label null. Panels were transferred to GSE30528 and GSE142025. **Results:** Panels contained 2.10–2.70 genes. Harmonic initialization improved hypervolume over Bernoulli and matched existing sparse initializers. Outer area under the curve (AUC) was 0.973–0.991, comparable to top-DEG and regularized selectors; permuted labels gave 0.510. Real GO raised maximum archive coherence from 0.044 to 0.960, and a GO-aware rule raised representative coherence from 0.098 to 0.620 at equal AUC. External AUC was 0.612–0.726 and 0.849–0.964. **Conclusions:** Harmonic initialization yields compact Pareto fronts without a size cap, GO guidance supplies coherent panels at equal discrimination, and regularized selection gives the most reproducible panel.

## 1. Introduction

Diabetic nephropathy, abbreviated DN, is a major cause of chronic kidney disease and kidney failure. Human kidney transcriptome studies have identified inflammatory, complement, extracellular-matrix, vascular, metabolic, and growth-factor programs associated with DN [1,2,3]. Integrated analyses have prioritized different genes across cohorts and analytical workflows [4,5], while a complement-focused study reported another disease-associated program [6]. Variation across disease stages and tissue compartments further complicates the discovery of compact and reproducible molecular panels.

These tissue programs sit downstream of systemic metabolic injury. Chronic hyperglycemia increases mitochondrial superoxide production and activates the polyol, hexosamine, protein kinase C, and advanced glycation end-product pathways, which converge on oxidative stress and inflammatory signaling in renal and vascular cells [7,8]. Innate immune pathways in immune cells and resident kidney cells then trigger and sustain renal inflammation [9]. Cellular antioxidant defense depends partly on selenium-containing enzymes, and selenoproteins also modulate insulin signaling and glucose metabolism [10]. Insulin signaling is further restrained by protein tyrosine phosphatases such as PTP1B [11], and lipid accumulation adds lipotoxic stress to podocytes and tubular cells [12]. A kidney transcriptome therefore mixes kidney-specific responses with signals of broader metabolic disturbance. A small panel that separates DN tissue from control tissue may capture either component.

Kidney gene-expression matrices contain many more features than samples. Correlated expression, platform differences, and adaptive evaluation of many candidate panels can therefore yield optimistic performance estimates and unstable signatures. Leakage occurs when information from a nominal validation sample influences differential-expression screening or any later model-selection step [13,14]. Feature screening must consequently be repeated within training data for every held-out evaluation. A high internal fitness value alone does not establish generalization, especially after the candidate pool has been enriched for differential expression.

Multi-objective evolutionary algorithms, abbreviated MOEAs, can retain trade-offs among discrimination, panel size, and biological structure without assigning a single weighted score. A practical difficulty is that standard Bernoulli initialization produces chromosomes containing roughly half of all candidate genes. A hard panel-size limit avoids this dense starting population but makes the final search space depend on an externally chosen bound. Large-scale sparse MOEAs address the same difficulty with score-guided initialization and sparsity-aware operators; SparseEA is the best-known example [15]. We use a harmonic sparse initializer that favors small panels while assigning positive probability to every nonempty panel size. Crossover and mutation subsequently operate without a cardinality repair rule. An NSGA-II ablation compares stratified harmonic sampling with independent and identically distributed (IID) harmonic draws, expected-size-matched independent Bernoulli sampling, standard dense Bernoulli sampling, a bounded uniform initializer used in earlier biomarker MOEAs [16], the SparseEA initializer, and the complete SparseEA algorithm.

Gene Ontology (GO) [17] can provide biological search guidance. Because the same annotation source drives selection, we treat GO coherence as an explicit third objective and as a target-attainment outcome. A structured randomized-GO control preserves annotation-set sizes and gene annotation counts while breaking the connection between gene identity and annotation. A two-objective control removes GO entirely. Coherence under Reactome, KEGG, and MSigDB Hallmark annotations, which were not used during search, provides a separate check of whether GO guidance carries over to other curated pathway resources.

The study uses repeated nested evaluation in GSE96804. Differential-expression screening, candidate-pool construction, evolutionary search, and representative-panel selection occur within each outer training split. In the final nested experiment, the corresponding outer fold is held out during screening, search, and final model fitting until evaluation with training-derived standardization. Six MOEAs are compared under real-GO three-objective optimization using the same termination target and paired outer splits. After exploratory development and before the corrected formal run, NSGA-II was designated as the reference algorithm for the two GO controls. Same-size random panels and a top differentially expressed gene baseline, denoted top-DEG, test whether evolutionary search adds value beyond chance or simple marginal ranking. LASSO, elastic-net, recursive feature elimination, and stability-selection baselines are evaluated on the same splits [18,19,20,21].

Gene sets are subsequently examined in GSE30528 and GSE142025. Two transfer analyses are reported. In cohort-specific refitting, classifiers are fitted separately within each external cohort, so this analysis assesses feature-set portability. In locked transfer, the coefficients and threshold learned in GSE96804 are applied unchanged after label-free standardization within each external cohort. In all three cohorts, DN kidney tissue is compared with non-DN kidney tissue.

The primary contribution of this work is an evaluation protocol for evolutionary gene-panel discovery and the empirical answers it gives in a small DN cohort. Its compon- ents are as follows:

- A within-run leakage-controlled evaluation protocol that repeats supervised screening and panel search within outer training data, together with repeated search seeds, an extended set of outer repeats, a permuted-label null, and inner-fold screening as robustness checks.
- A harmonic sparse initialization method that covers every nonempty panel size without enforcing a size limit after initialization, evaluated against IID harmonic, two Bernoulli, bounded uniform, and SparseEA initializers and the complete SparseEA algorithm; the harmonic law improves search efficiency relative to Bernoulli initialization.
- A real-GO three-objective formulation with two structured NSGA-II controls that distinguish biological search guidance from generic third-objective effects, checked against annotation resources not used during search.
- A paired comparison of six MOEAs with random, top-DEG, and established embedded and wrapper feature-selection baselines, followed by refitted and locked cross- cohort analyses.

The remainder of this article is organized as follows. Section 2 reviews DN transcriptomics, validation bias, evolutionary feature selection, and biological optimization objectives. Section 3 describes the datasets, nested evaluation, harmonic sparse initialization, initialization and GO ablations, comparator baselines, robustness analyses, statistical analysis, and external transfer. Section 4 reports the internal outer-fold comparisons, Pareto-front characteristics, initialization and GO ablations, robustness analyses, feature recurrence, and cross-cohort results. Section 5 discusses the findings, scientific limitations, and priorities for further validation. Section 6 summarizes the conclusions.

## 2. Related Work

### 2.1. DN Transcriptomics and Molecular Heterogeneity

Human kidney transcriptome studies have reported compartment-specific changes in Rho-family GTPase, integrin, vascular endothelial growth factor, inflammatory, and complement programs [1], and whole-biopsy RNA sequencing linked immune-response and fibrosis genes to advanced DN and lower estimated glomerular filtration rate (eGFR) [2]; in microdissected glomeruli, early DN mainly altered metabolic processes, whereas late DN added immune, complement, and extracellular-matrix pathways [3]. Integrated bioinformatics studies have proposed hub-gene signatures containing VEGFA, NPHS1, WT1, CTGF, SYNPO, and PODXL [4] or FN1, C1QA, C1QB, and CD44 [5]. A complement-focused analysis prioritized C1QA, C1QB, C3, CFB, ITGB2, VSIG4, and CLU [6]. Differences among signatures may reflect cohort composition, disease stage, tissue compartment, preprocessing, and selection criteria. Multi-omics and mechanistic reviews likewise emphasize that DN is not represented by one universal molecular program [22,23].

### 2.2. Machine Learning and Validation Bias

Network-based studies have combined differential expression, weighted gene co-expression network analysis (WGCNA), protein-interaction networks, and receiver operating characteristic (ROC) or immune-infiltration analysis to identify candidate markers [24,25]. Related work incorporated immune and oxidative-stress features with machine learning [26]. A recent systematic review reported recurring limitations that include small samples, heterogeneous predictors, incomplete development reporting, and limited external validation [27]. These concerns become acute when investigators perform feature screening before cross-validation. Nested evaluation is required when the reported performance is intended to cover the complete discovery procedure [13,14].

Artificial intelligence has also been applied to the detection of other diabetic complications. Deep learning detects referable diabetic retinopathy from fundus photographs with high sensitivity and specificity [28], and machine-learning models estimate diabetic kidney disease (DKD) risk from clinical, laboratory, retinal-imaging, and metabolomic variables [29,30]. These settings differ from the present one. Imaging and electronic-record studies can draw on thousands of patients and on inputs available at the point of care, whereas tissue transcriptomes require a biopsy or nephrectomy, come from tens of samples per cohort, and change with assay platform. Transcriptomic panel discovery therefore operates in a regime where adaptive search can overfit easily, and where interpretability and assay size matter more than marginal gains in internal discrimination.

### 2.3. Multi-Objective Evolutionary Feature Selection

Evolutionary feature selection can search interacting feature subsets while retaining trade-offs between prediction and panel size. Pareto optimization has been used to identify diverse molecular signatures [31]. Subsequent biomarker-selection studies traded predictive performance against panel size [16] and added fold-change separation and survival prediction as further objectives [32]. MEvA-X jointly tunes XGBoost and feature selection, while DOSA-MO explicitly distinguishes internal fitness from left-out performance [33,34]. Sparse initialization has commonly been implemented through a bounded number of selected features, a prescribed sparsity interval, or specialized sparse operators. Cattelani and Fortino drew each initial subset size uniformly between zero and 50 [16]. For large-scale sparse multi-objective problems, SparseEA first evaluates every single-variable solution, uses the resulting nondominated ranks as variable scores, and builds initial masks through score-based binary tournaments with a uniformly random number of draws; its crossover and mutation then add or remove variables guided by the same scores [15]. Later algorithms mine the sparse distribution of nonzero variables during evolution [35]. These approaches reduce the dense-start problem but introduce a cardinality range or continue to regulate sparsity during evolution. The present initializer instead defines a probability distribution over all nonempty cardinalities and does not modify panel size after initialization. It requires no scoring evaluations and no problem-specific operators, so it can be paired with any binary MOEA, and the ablation in this study compares it directly with the bounded uniform and SparseEA initializers and with the complete SparseEA algorithm.

### 2.4. Biological Knowledge as an Optimization Target

Prior biological knowledge has guided multi-objective module identification and gene clustering [36,37]. Graph structure and multiple data views have also been incorporated into multi-objective gene selection [38,39], while related evolutionary methods have targeted disease modules and protein complexes [40,41]. Over-representation tools support functional interpretation of selected gene sets [42,43], but conclusions depend on the background universe, annotation redundancy, gene correlation, and multiplicity; gene dependence is a particular limitation of simple enrichment analysis [44]. When GO relevance drives selection, a favorable GO score after selection is conditional on that mechanism. It demonstrates target attainment but does not provide independent biological confirmation. A meaningful ablation should therefore compare real annotation with both removal of the objective and a structure-preserving randomized annotation target.

### 2.5. Study Motivation

The reviewed literature leaves three linked research gaps. First, existing evolutionary biomarker studies demonstrate the value of multi-objective feature selection, but sparsity is commonly coupled to a permitted cardinality range or to operators that continue to regulate subset size. This coupling does not isolate whether sparse coverage at initialization alone improves finite-budget search, and it requires a user-defined size range. Second, predictive performance can be optimistic when differential-expression screening, adaptive panel search, or representative selection precedes the evaluation split. An evaluation that nests the complete discovery process and includes same-size random and top-DEG controls is needed to distinguish evolutionary search from chance subsets and simple marginal ranking. Third, when biological annotation is an optimization objective, a favorable annotation score is expected by design and cannot serve as independent biological validation. Removal and randomized-annotation controls are therefore needed to determine whether real annotation changes search behavior or held-out prediction.

This study addresses these gaps with harmonic sparse initialization, which distributes initial solutions across every nonempty cardinality and then leaves panel size unconstrained during crossover and mutation. Repeated outer cross-validation nests candidate screening, three-objective optimization, and representative-panel selection. Six algorithms are compared under the same formulation, and their held-out panels are benchmarked against matched random and top-DEG controls and established feature-selection methods. Separate NSGA-II ablations compare initialization laws and remove or randomize the GO objective. Cohort-specific refitting and locked transfer in GSE30528 and GSE142025 then assess feature-set portability and the transfer of fixed models. This design separates four questions: whether initialization alone improves access to compact Pareto fronts, whether evolutionary panels outperform simple controls, whether GO provides genuine search guidance, and whether the selected feature sets transfer across cohorts.

## 3. Materials and Methods

This section follows the analysis from cohort assignment to reproducible evaluation. Section 3.1 defines the cohort roles, and Section 3.2 describes leakage-controlled outer resampling and split-specific screening. Section 3.3, Section 3.4, Section 3.5 and Section 3.6 specify the objectives, harmonic initialization, its ablation, the evolutionary algorithms, and representative-panel selection. Section 3.7, Section 3.8, Section 3.9, Section 3.10 and Section 3.11 define the GO controls, search-front indicators, matched baselines, robustness analyses, and statistical inference. Section 3.12 and Section 3.13 cover external refitting and locked transfer, and reproducibility.

### 3.1. Study Design and Datasets

**Figure 1:**
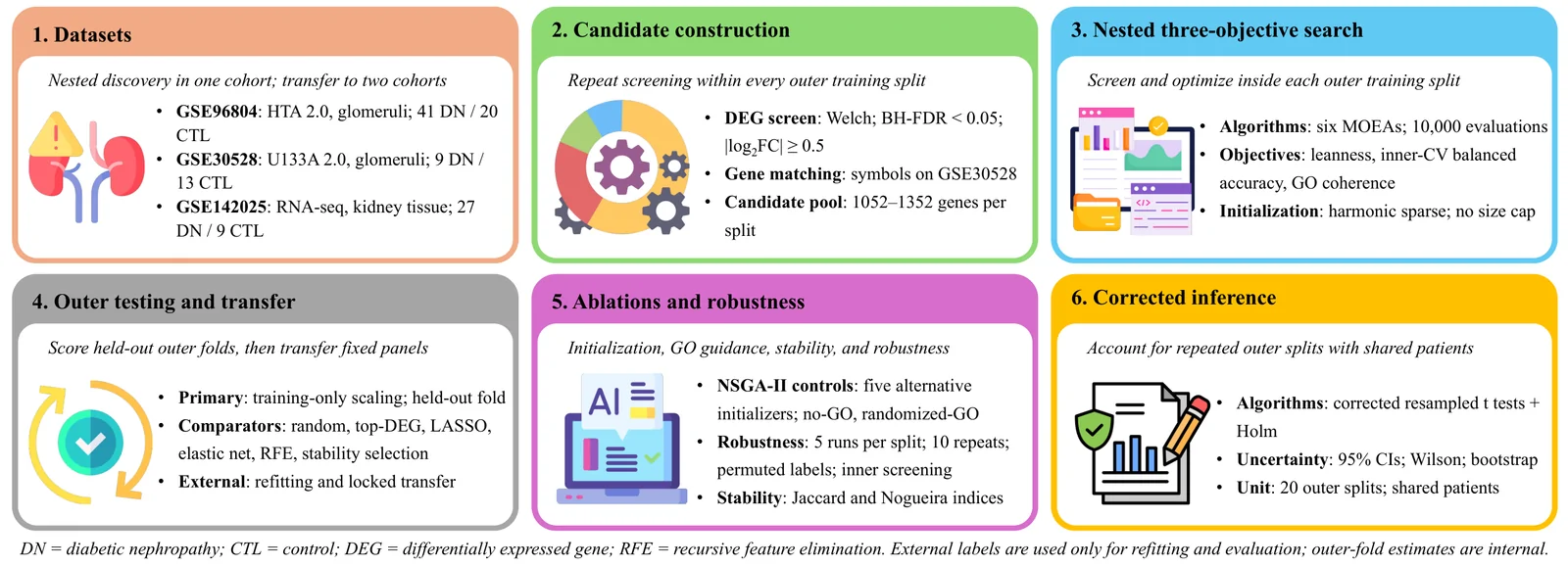
Within-run leakage-controlled study design for the final nested runs. GSE96804 underwent four repetitions of stratified five-fold outer cross-validation. DEG screening, candidate-pool construction, harmonic sparse initialization, evolutionary search, and representative-panel selection were confined to each outer training split. All six algorithms used the real-GO three-objective formulation; NSGA-II additionally underwent no-GO, randomized-GO, and five initialization-control ablations, and the complete SparseEA algorithm was added for comparison. Established feature-selection methods and the robustness runs used the same outer splits. Outer folds held out within each final run supplied the internal performance estimates, and fixed panels were examined by cohort-specific refitting and locked transfer in GSE30528 and GSE142025. BH-FDR, Benjamini–Hochberg false discovery rate; CI, confidence interval; CTL, control; CV, cross-validation; DEG, differentially expressed gene; DN, diabetic nephropathy; GO, Gene Ontology; HTA, Human Transcriptome Array; MOEA, multi-objective evolutionary algorithm; RFE, recursive feature elimination.

All three cohorts were obtained from the NCBI Gene Expression Omnibus [45]. GSE96804 was the discovery cohort and supplied the repeated outer evaluation. GSE30528 gene coverage restricted the search space to genes measurable on that platform; its expression values and labels did not enter screening or optimization. GSE142025 was not used in candidate-pool construction. Fixed panels from the nested experiment were subsequently examined in both external cohorts without pooling cohorts. Table 1 summarizes the cohort roles, and Figure 1 shows the complete analysis flow.

### 3.2. Outer Evaluation and Candidate-Pool Construction

Processed series matrices were analyzed as supplied, and cohorts were never pooled or batch-corrected together. Platform annotations were mapped to HUGO Gene Nomenclature Committee (HGNC) gene symbols. Multiple probes or transcript clusters mapping to the same symbol were averaged on the processed log scale. GSE96804 underwent four repetitions of stratified five-fold outer cross-validation with random state 20260920, producing 20 outer train–test splits. Within each final nested run, the outer fold remained unavailable during every supervised discovery step.

Within each outer training split, differential expression was tested by Welch’s two-sample *t* test. *p*-values received Benjamini–Hochberg adjustment [46]. Genes required BH-FDR <0.05 and absolute mean log_2_ fold change ≥0.5. Retained genes were intersected with GSE30528 gene coverage and capped at 2000 by unadjusted *p*-value rank. The candidate pool was rebuilt for every outer split and ranged from 1052 to 1352 genes. Its mean size was 1205.8, and the largest pool, with 1352 genes, stayed below the 2000-gene cap, which was therefore never active. This restriction used only assay availability from GSE30528.

After panel selection, an L2-regularized standardized logistic-regression classifier with fixed C=0.1 was fitted to the complete outer training split. The scaler was fitted on outer training samples and applied unchanged to the outer test samples. The area under the ROC curve (AUC), with DN as the positive class, and balanced accuracy at threshold 0.5 were calculated once on that outer test split. The 20 outer results form the primary internal performance evidence. Sensitivity and specificity at the same threshold were also recorded. To quantify uncertainty at the patient level, each GSE96804 sample received the mean of its four out-of-fold probabilities, one per outer repeat; the sensitivity and specificity of these averaged probabilities are reported with Wilson 95% intervals.

### 3.3. Three-Objective Formulation

Let $P=\{g_{1},\ldots ,g_{m}\}$ be the split-specific candidate pool and let S⊆P be a nonempty panel. The main experiment maximized

(1) $$\begin{array}{cc} f_{1}\left(S\right) & =\frac{1}{|S|+1},\\ f_{2}\left(S\right) & =\frac{1}{5}\sum_{k=1}^{5}{\mathrm{BA}}_{k}\left(S\right),\\ f_{3}\left(S\right) & =\frac{2}{\left|S|(|S|-1\right)}\sum_{\begin{array}{c} g_{i},g_{j}\in S\\ i<j \end{array}}q_{ij}. \end{array}$$

Here, $f_{1}$ rewards compact panels and $f_{2}$ is the mean balanced accuracy from stratified five-fold cross-validation within the outer training split. Each inner fold fitted its own standardization and logistic-regression model with C=0.1. The optimizer minimized $\left(-f_{1},-f_{2},-f_{3}\right)$.

The biological objective $f_{3}$ measures GO Biological Process coherence. GO terms were restricted to the split-specific candidate universe. Terms containing fewer than three candidate genes or more than half of that universe were excluded. For two candidate genes, $q_{ij}$ is the Jaccard similarity between their retained GO-term membership sets,

(2) $$q_{ij}=\frac{|T_{i}\cap T_{j}|}{|T_{i}\cup T_{j}|},$$

with zero assigned when the union is empty. The score averages over all distinct gene pairs and remains in [0,1]. Under random panel sampling, its expectation is the mean pairwise similarity of the candidate universe and therefore does not increase mechanically with panel cardinality. Panels containing one gene receive $f_{3}=0$.

Candidate-pool screening used the complete outer training split before the inner folds were formed. Consequently, $f_{2}$ serves as search fitness, and Section 3.10 describes a robustness analysis that repeated the screen inside every inner training fold. The corresponding held-out outer fold evaluates the complete discovery procedure within each final nested run. Similarly, $f_{3}$ is an optimized target and its attained value describes GO-guided search behavior.

### 3.4. Harmonic Sparse Initialization

High-dimensional binary feature selection creates an initialization-scale problem. For *m* candidate genes, independent Bernoulli(0.5) sampling concentrates the initial cardinality near m/2, so much of a finite evaluation budget may be spent moving from dense solutions toward compact panels. Sampling cardinality uniformly from 1,…,m removes that concentration but assigns only a small finite-population share to the smallest sizes, whereas imposing an upper bound introduces a user-chosen panel-size assumption. Because leanness is already an optimization objective, our aim was to bias the initial population toward sparse panels while preserving access to every nonempty cardinality.

The harmonic law provides this compromise. Because $\sum_{k=a}^{\left\lfloor ca\right\rfloor }1/k\approx \log c$, multiplicative cardinality ranges receive approximately comparable probability mass after normalization; small absolute sizes are emphasized without excluding larger panels. Each panel was represented by a binary vector $x\in {\left\{0,1\right\}}^{m}$, and its initial cardinality *K* was sampled from

(3) $$\mathrm{Pr}\left(K=k\right)=\frac{1}{kH_{m}},\;H_{m}=\sum_{r=1}^{m}\frac{1}{r},\;k=1,\ldots ,m.$$

Conditional on K=k, the initializer selected *k* genes uniformly without replacement. Stratified inverse-transform quantiles were used within each population so that the finite initial population covered the harmonic distribution more evenly than independent draws. Every nonempty panel size had positive probability, while small panels received more initial mass.

**Figure 2:**
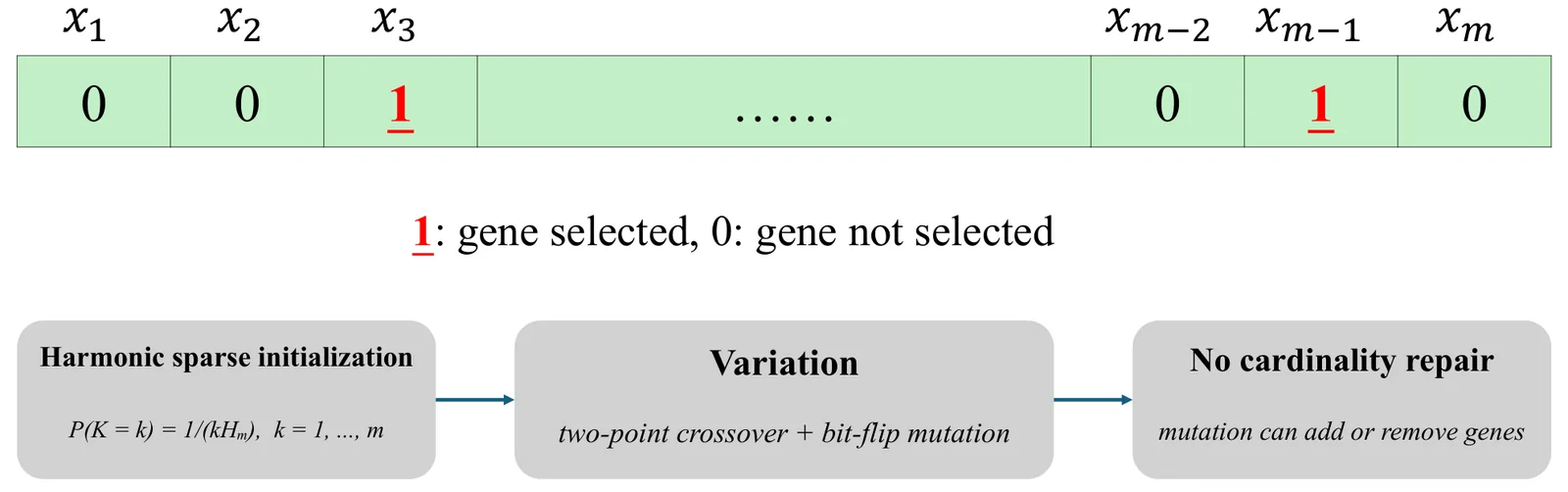
Binary panel encoding and harmonic sparse initialization. Each bit corresponds to one candidate gene, and a value of 1 includes that gene. The initial cardinality follows $\mathrm{Pr}\left(K=k\right)=1/\left(kH_{m}\right)$. Crossover and mutation subsequently operate without a panel-size cap or repair operator.

No panel-size upper bound, lower bound beyond nonemptiness, or cardinality repair was applied after initialization. Two-point crossover and bit-flip mutation could add or remove selected genes. An empty chromosome generated by variation received the worst objective vector and therefore could not become a valid nondominated panel. Figure 2 illustrates the encoding and search operators.

### 3.5. Initialization Ablation

The NSGA-II real-GO condition was used to isolate three components of the proposed initializer. First, an IID harmonic control drew each cardinality independently from the same $\mathrm{Pr}\left(K=k\right)=1/\left(kH_{m}\right)$ law, removing only the stratified inverse-transform quantiles. Second, an expected-size-matched Bernoulli control sampled

(4) $$x_{j}∼\mathrm{Bernoulli}\left(1/H_{m}\right),$$

so that $E\left[K\right]=m/H_{m}$, equal to the harmonic expectation, while concentrating cardinality around its mean. Third, standard dense random initialization used $x_{j}∼\mathrm{Bernoulli}\left(0.5\right)$. An all-zero Bernoulli draw was changed to one randomly selected gene only at initialization.

Three existing sparse strategies were added during revision. The bounded uniform initializer drew each initial panel size uniformly from 1 to 50, following the scheme of Cattelani and Fortino [16], whose range starts at zero; the empty panel was excluded because it cannot be evaluated, and no cap was applied after initialization. The SparseEA initializer evaluated all *m* single-gene panels, scored each gene by the nondominated front of its single-gene solution, and built each initial panel by ⌊um⌋ binary tournaments on these scores, with u∼U(0,1) [15]. It was combined with the same NSGA-II operators. The complete SparseEA algorithm used the same initializer together with its score-guided mask crossover and mutation and NSGA-II environmental selection [15]. Because binary panel selection has no real-valued decision vector, only the mask component of SparseEA was used. The *m* single-gene evaluations were counted against the 10,000-evaluation budget for both SparseEA conditions.

The stratified harmonic arm reused the 20 completed NSGA-II real-GO runs. The 60 original control runs and the 60 runs added during revision were executed separately. All seven conditions used identical outer splits, split-specific candidate pools, search seeds, population size, crossover, mutation, 10,000-evaluation target, objectives, and representative rule. None used a size cap or cardinality repair during evolution. Final fronts were normalized jointly across the seven initialization conditions within each outer split. Hypervolume (HV) used reference point (1.1,1.1,1.1), and empirical inverted generational distance (IGD) used the nondominated union of their normalized final fronts. For each of five outcomes—HV, IGD, outer AUC, outer balanced accuracy, and representative size—the six contrasts with stratified harmonic sampling received Holm correction.

### 3.6. Multi-Objective Evolutionary Algorithms and Representative Panels

Six algorithms were evaluated under the real-GO three-objective formulation: NSGA-II and its balanced tie-breaking variant [47,48], SPEA2 and SPEA2+ [49,50], and MOEA/D and MOEA/D-NRD [51,52]. Every run used a target of 10,000 fitness evaluations. The population size was 100 for the NSGA-II and SPEA2 families. The three-objective MOEA/D algorithms used the smallest Das–Dennis reference-direction set containing at least 100 directions, giving 105 subproblems. Two-point crossover had probability 0.9, and bit-flip mutation had per-bit probability 1/m. Table 2 summarizes the settings.

For each final nondominated archive, the representative panel had the largest inner-CV balanced accuracy. Greater leanness and then archive order resolved ties. The same rule was used for every condition and did not inspect the GO objective. This GO-blind rule separates predictive representative selection from archive-level GO target attainment.

To show how a practitioner can use the GO dimension of the archive, a GO-aware tolerance rule, defined after the main analysis, was applied to the final archives as a sensitivity analysis. Within each final archive, panels whose inner-CV balanced accuracy lay within δ of the archive maximum were retained, and the panel with the largest real-GO coherence was chosen; smaller size and then higher inner accuracy resolved ties. We report δ=0.025, which corresponds to about one misclassified training sample across the five inner folds, and δ=0.05. The chosen panels were refitted and scored on the outer test fold exactly as the accuracy-first panels.

### 3.7. GO-Objective Ablation

The main real-GO condition was run for all six algorithms. After exploratory development and before the corrected formal run, NSGA-II was designated as the reference algorithm for the ablation. The first control removed $f_{3}$ and optimized only $f_{1}$ and $f_{2}$. The second retained three objectives but randomized the relationship between gene identity and GO annotation. Within each outer split, complete gene annotation profiles were permuted among candidate genes using seed 20263000+s for split *s*. This permutation preserved every retained GO term size, the distribution of annotation counts per gene, and the complete pairwise-similarity distribution. It removed the biological correspondence between a gene and its annotation profile. The three NSGA-II conditions used identical outer splits, search seeds, operators, initialization, population size, and evaluation target.

Panels and final archives from all conditions were rescored with the real-GO function. Representative-panel coherence and the archive maximum, mean, and 90th percentile quantified target attainment on a common scale. These summaries test whether real annotation guidance produces greater real-GO coherence than removing GO or optimizing a structurally matched random annotation target.

Three annotation resources that were not used during search provided a separate check: Reactome 2022 [53], KEGG 2021 Human [54], and MSigDB Hallmark 2020 [55], all obtained as Enrichr libraries [56]. For each resource, pairwise Jaccard coherence was computed exactly as for GO, with the same split-specific term filters. We report the archive maximum of each coherence score and the coherence of representative and tolerance-rule panels relative to the mean of 200 random candidate-pool panels of the same size. Genes that appeared in any real-GO representative panel were also tested for over-representation in these resources against the union of the 20 candidate pools as background, using the hypergeometric test with Benjamini–Hochberg adjustment within each resource. Because GO and these pathway resources overlap in content, this analysis checks consistency across annotation sources.

### 3.8. Search-Front Descriptors

For a nondominated set *A* in a *d*-objective minimization space and a reference point r dominated by every solution, hypervolume is

(5) $$\mathrm{HV}\left(A;r\right)=\lambda_{d}\;\left(\underset{a\in A}{⋃}\prod_{j=1}^{d}\left[a_{j},r_{j}\right]\right),$$

where $\lambda_{d}$ is *d*-dimensional Lebesgue measure. Larger HV indicates a larger dominated region toward preferred objective values. For an empirical reference front $R_{\mathrm{emp}}$, inverted generational distance is

(6) $${\mathrm{IGD}}_{\mathrm{emp}}\left(A,R_{\mathrm{emp}}\right)=\frac{1}{|R_{\mathrm{emp}}|}\sum_{z\in R_{\mathrm{emp}}}\min_{a\in A}{∥z-a∥}_{2}.$$

Lower IGD indicates closer coverage of the empirical reference front. Candidate pools differed across outer splits, so front descriptors were calculated within matched splits.

### 3.9. Random and Top-DEG Controls

Each representative panel was compared on the same outer test split with 50 random panels sampled from that split’s candidate pool. Random panels matched the representative panel size. Their metrics were averaged within the split. The deterministic top-DEG control used the first |S| genes from the split-specific differential-expression ranking. It therefore matched the evolutionary panel size and used no outer-test label information.

Five established feature-selection methods were added as comparators. Each was applied to the standardized expression of the same split-specific candidate pool in the outer training split only. LASSO logistic regression [18] chose its penalty from 20 values between $10^{-2}$ and $10^{2}$ by stratified five-fold inner CV on ROC-AUC. Elastic-net logistic regression [19] chose its penalty from ten values between $10^{-4}$ and $10^{4}$ and the mixing parameter from 0.1, 0.5, and 0.9 by the same inner CV. Recursive feature elimination [20], RFE, used the logistic-regression classifier with C=0.1 and removed 10% of the remaining genes per step; one version stopped at the NSGA-II panel size of the same split and a second, RFECV, chose the size by inner CV on balanced accuracy. Stability selection [21] fitted L1 logistic regression to 100 stratified half-samples at five penalties between 0.05 and 1.0 and kept genes whose maximum selection probability was at least 0.6; when no gene reached this threshold, the most frequently selected gene was kept. The selected genes were then scored on the outer test fold with the same standardized L2 logistic-regression classifier used for the evolutionary panels, so the comparison concerns gene selection and not the final classifier.

### 3.10. Robustness Analyses

Five additional analyses addressed sources of variability and optimism. First, every algorithm was run four more times on each of the 20 outer splits with different evolutionary seeds, 20261000+s+1000r for run r=1,…,4, while the inner-CV partition was kept fixed; this gave five independent runs per algorithm and split. Second, the outer cross-validation was extended from four to ten repeats with the same random state, which reproduces the original 20 splits and adds 30 new ones, and all six algorithms were run on the new splits. Third, the complete NSGA-II pipeline was run on 20 random permutations of the GSE96804 labels for the five outer folds of the first repeat. The FDR screen returned no gene under permuted labels, so the candidate pool for this null analysis consisted of the 1206 genes with the smallest Welch P-values, which equals the mean size of the real pools. Fourth, all six algorithms were rerun with the differential-expression screen repeated inside every inner training fold: for each inner fold, panel genes that did not pass the screen on that fold’s training samples were removed before the inner classifier was fitted, and a fold without remaining genes scored 0.5. Fifth, NSGA-II was rerun without restricting the candidate pool to genes measured on GSE30528. In that setting, 2265 to 2917 genes passed the screen, so the 2000-gene cap became active.

### 3.11. Statistical Analysis

Because the 20 outer results reuse patients across repetitions, paired contrasts used the Nadeau–Bengio corrected resampled *t* approximation [57]. For paired differences $d_{1},\ldots ,d_{n}$, the statistic was

(7) $$t_{\mathrm{corr}}=\frac{\bar{d}}{\sqrt{\left(\frac{1}{n}+\frac{n_{\mathrm{test}}}{n_{\mathrm{train}}}\right)s_{d}^{2}}},$$

with n−1 degrees of freedom. Here, n=20 and the mean correction factor was 0.3001; analyses added during revision used the ratio of mean fold sizes, 12.2/48.8, giving 0.3000 with n=20 and 0.2700 with n=50 for the ten-repeat extension. All 15 main-experiment algorithm pairs received Holm correction separately for outer AUC, balanced accuracy, panel size, real-GO coherence, HV, and IGD. Comparisons with random and top-DEG controls received Holm correction across six algorithms for each metric and control. The two NSGA-II GO-ablation contrasts, real GO versus no GO and real GO versus randomized GO, received Holm correction within each representative or archive-level metric. Initialization contrasts received Holm correction across the six comparisons with stratified harmonic sampling separately for each of the five outcomes. External AUC and balanced-accuracy contrasts were analyzed across the 20 discovery-split panels with the same corrected paired approximation and Holm correction across six algorithms within each cohort, metric, and control. Because every panel was evaluated on the same external patients, these external *p*-values quantify consistency across panels within the observed cohorts. Paired differences in Table 3, for the GO-aware rule, and in Supplementary Tables S3 and S6 are reported with 95% confidence intervals from the same corrected variance. Selection stability across the 20 outer splits was summarized by the mean pairwise Jaccard similarity of selected gene sets and by the stability estimator of Nogueira et al. [58], which corrects for panel size and for the size of the candidate universe.

### 3.12. External Cohort-Specific Refitting

The fixed panels were evaluated separately in GSE30528 and GSE142025. Within each cohort, stratified five-fold cross-validation produced one out-of-fold probability for every sample. Each fold fitted its scaler and logistic-regression coefficients using only that fold’s training samples. Genes absent from a cohort were removed before fitting, and effective panel size was recorded. Fifty random panels and one top-DEG panel matched each effective size and used the same folds. Both controls were drawn from the corresponding discovery-split candidate pool after restricting it to genes available in the external cohort; the top-DEG order came from that discovery split, and no external labels were used to rank genes.

The gene identities were fixed before external labels were used, but classifier coefficients were refitted within each external cohort. These results therefore assess feature-set portability. Sensitivity and specificity were recorded at the 0.5 threshold, and, as a sensitivity analysis, at a threshold equal to the case proportion of each training fold.

A locked-model analysis was added during revision. For each of the 20 discovery splits, the scaler, logistic-regression coefficients, and 0.5 threshold fitted on the GSE96804 outer training split were applied unchanged to each external cohort. The three assays measure expression on different scales, so each gene was z-scored within the external cohort before the locked coefficients were applied. This step uses no external labels. We report AUC, sensitivity, and specificity for each locked split model and the AUC of a locked ensemble that averages the 20 split-model probabilities per external patient, with a 95% interval from 2000 stratified bootstrap resamples.

### 3.13. Software and Reproducibility

The outer resampling seed was 20260920. Evolutionary searches used seed 20261000+s for outer split *s*, so algorithms and conditions shared the same split-specific seed. Random-panel controls used a separate deterministic sequence beginning at 20262000, and randomized GO used the sequence beginning at 20263000. In the primary analysis each algorithm was executed once per outer split; the four additional runs per split described in Section 3.10 estimate stochastic algorithm variability separately from split variability. Source code, task-level metadata, representative panels, and complete final nondominated archives were retained for every run. Analyses used Python 3.14.6 with NumPy 2.5.3, SciPy 1.18.1, pandas 3.0.6, scikit-learn 1.9.1, pymoo 0.6.2, statsmodels 0.15.0, and GEOparse 2.0.4. The GO annotation was the Enrichr GO_Biological_Process_2023 library. Supplementary Section S8 lists all seeds, software versions, and gene-set file checksums, and the split-level results of every analysis are provided in the Supplementary Data file. Information for accessing the original datasets and analysis code is provided in the Data Availability Statement.

## 4. Results

This section presents the findings in the order of the analytical questions. Section 4.1 and Section 4.2 describe the nested candidate pools, emergent panel sizes, and held-out internal performance, and Section 4.3 compares the panels with established feature-selection methods. Section 4.4 compares the three-objective search fronts. Section 4.5 and Section 4.6 isolate the effects of the initialization law and the GO objective. Section 4.7 reports the robustness analyses. Section 4.8 and Section 4.9 then assess feature stability and cross-cohort transfer.

### 4.1. Nested Candidate Pools and Emergent Panel Size

The 20 outer training splits produced candidate pools of 1052–1352 genes, with a mean of 1205.8. Within each final nested run, the corresponding outer fold contained 12 or 13 samples and remained unavailable during screening, optimization, and representative-panel selection.

Despite the absence of a cardinality bound or repair operator, the accuracy-first representative rule selected small panels. Mean panel size ranged from 2.10 for SPEA2+ to 2.70 for MOEA/D-NRD. Mean inner-CV balanced accuracy was 1.000 for NSGA-II, NSGA-II tie-break, SPEA2, and SPEA2+, 0.997 for MOEA/D, and 0.988 for MOEA/D-NRD. No pairwise panel-size difference remained significant after Holm correction. Mutation could add or remove genes, so the small representative panels arose from selection pressure after inner fitness had saturated; neither a deletion-only operator nor an imposed upper limit caused this result.

### 4.2. Internal Outer-Fold Performance

**Figure 3:**
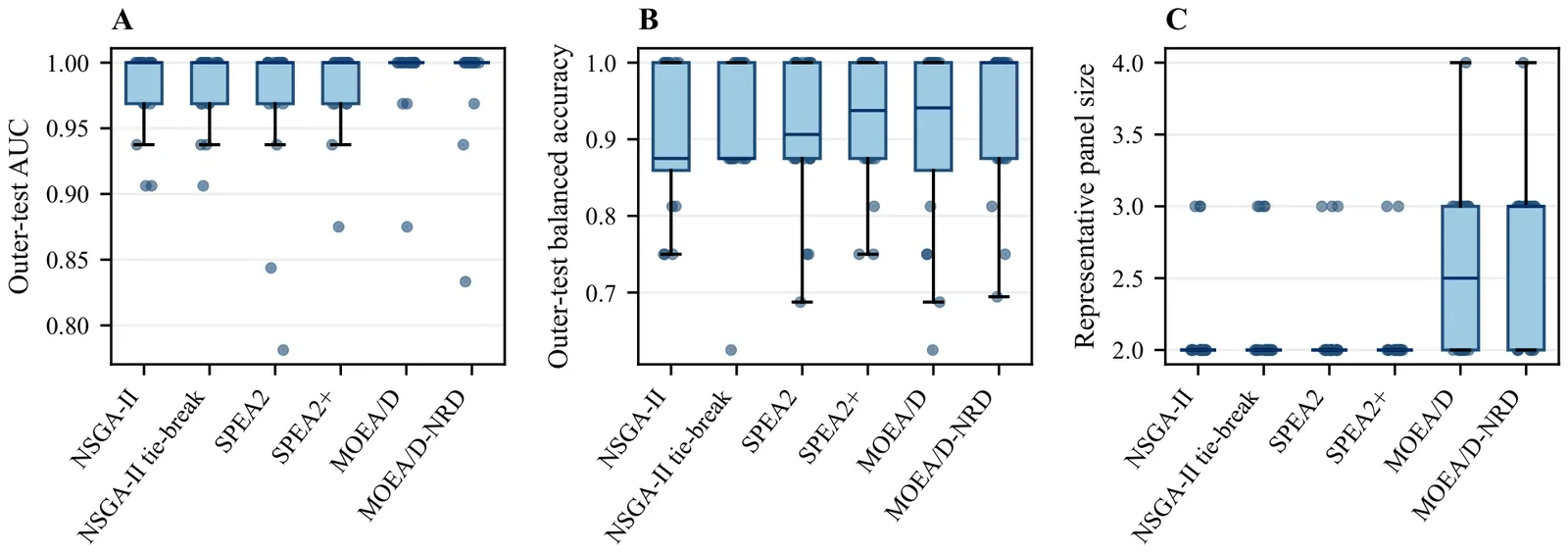
Outer-test performance and representative-panel size across 20 matched outer splits. Boxes summarize split-level distributions and dots show individual splits. Repeated splits share patients and are interpreted using corrected paired inference. (**A**) Outer-test AUC; (**B**) outer-test balanced accuracy; (**C**) representative panel size. AUC, area under the receiver operating characteristic curve; BA, balanced accuracy.

Table 3 presents the primary internal predictive results, and Figure 3 shows their split-level distributions. Mean outer-fold AUC ranged from 0.973 for SPEA2 to 0.991 for MOEA/D, and mean balanced accuracy ranged from 0.894 for NSGA-II to 0.932 for MOEA/D-NRD. None of the 15 pairwise algorithm contrasts was significant after Holm correction for AUC, balanced accuracy, or panel size. With out-of-fold probabilities averaged over the four outer repeats, every algorithm classified all 41 DN samples correctly, a sensitivity of 1.000 with a Wilson 95% interval of 0.914 to 1.000. Specificity ranged from 0.850 for NSGA-II, with an interval of 0.640 to 0.948, to 1.000 for both MOEA/D variants, with an interval of 0.839 to 1.000. The wide specificity intervals reflect the 20 control samples.

Four algorithms exceeded their split-matched random panels in AUC after correction: NSGA-II, NSGA-II tie-break, SPEA2+, and MOEA/D. NSGA-II, NSGA-II tie-break, SPEA2, and SPEA2+ also exceeded random panels in balanced accuracy. No algorithm exceeded the same-size top-DEG control after correction for either predictive metric; every adjusted AUC comparison with top-DEG had $p_{H}=1.000$. Evolutionary search therefore identified informative compact panels relative to random subsets of the screened pool and matched simple marginal DEG ranking.

### 4.3. Comparison with Established Feature-Selection Methods

Table 4 compares the evolutionary panels with five established methods applied to the same outer training splits and candidate pools. LASSO selected 4.85 genes on average and reached a mean outer AUC of 1.000 and a balanced accuracy of 0.950. Stability selection kept 1.15 genes with AUC 1.000, and RFE stopped at the NSGA-II panel size reached 0.998. Elastic net and RFECV retained 227.80 and 124.00 genes and classified every outer fold perfectly. Across the six algorithms, the outer AUC of the evolutionary panels was 0.008 to 0.027 lower than that of each baseline, and none of these differences was significant after Holm correction, with $p_{H}=1.000$ for every AUC contrast. Balanced accuracy was 0.068 to 0.106 lower than that of elastic net and RFECV, again without a corrected difference, with $p_{H}\geq 0.277$.

The baselines also selected far more reproducible gene sets. The mean pairwise Jaccard similarity of representative panels across the 20 outer splits was 0.013 to 0.050 for the six algorithms, compared with 0.120 for top-DEG, 0.329 for LASSO, 0.367 for size-matched RFE, and 0.866 for stability selection. The Nogueira stability index, which corrects for panel size and candidate-universe size, was 0.017 to 0.072 for the six algorithms and gave the same order. Standard embedded and wrapper selection therefore matched the evolutionary panels in discrimination and exceeded them in stability.

### 4.4. Three-Objective Search Fronts

SPEA2+ had the largest mean normalized hypervolume and the smallest mean empirical IGD, followed closely by NSGA-II tie-break; see Table 5. After correction across 15 pairs, NSGA-II, NSGA-II tie-break, SPEA2, and SPEA2+ each had larger HV than MOEA/D-NRD. No other HV contrast and no IGD contrast remained significant. The two decomposition methods retained substantially larger archives but covered less of the normalized empirical three-objective reference front under these settings.

**Figure 4:**
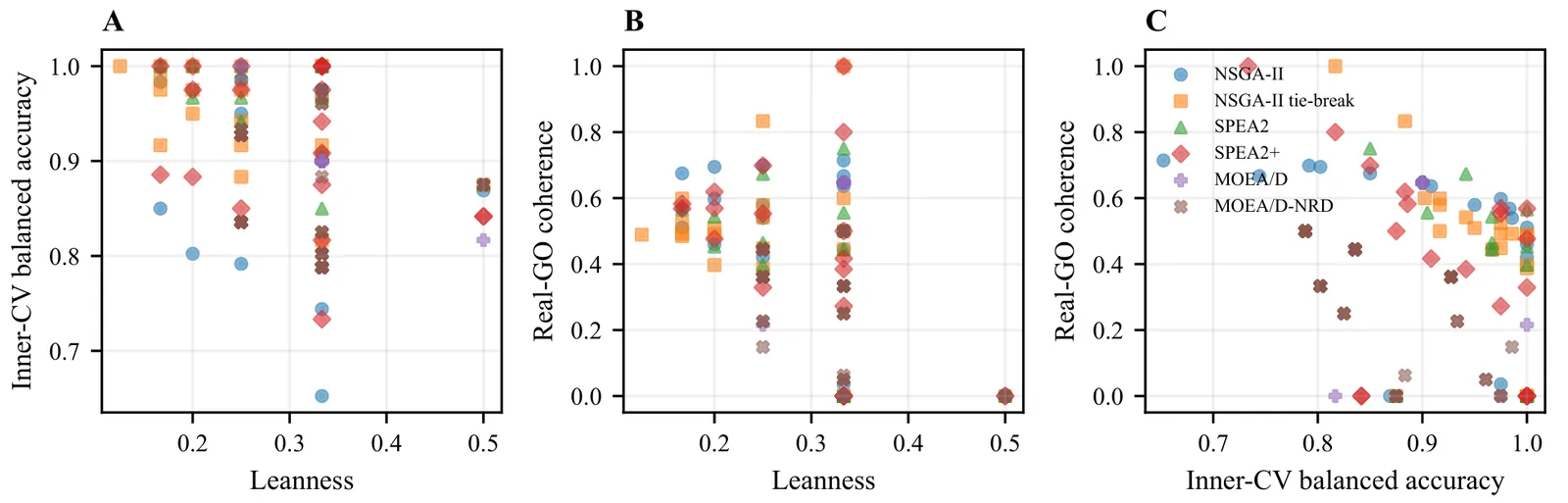
Pairwise projections of the real-GO three-objective nondominated sets for outer split 0. (**A**) Inner-CV balanced accuracy versus leanness; (**B**) real-GO coherence versus leanness; (**C**) real-GO coherence versus inner-CV balanced accuracy. Colors and markers identify the algorithms, as shown in the legend in panel C, and apply to all panels. Leanness equals 1/(|S|+1). The display describes the training search structure.

Figure 4 shows the three pairwise projections for outer split 0. Inner-CV balanced accuracy is a search-fitness value; the held-out outer-fold metrics in Table 3 provide the internal predictive evaluation of each final nested run.

### 4.5. Initialization Ablation Results

**Figure 5:**
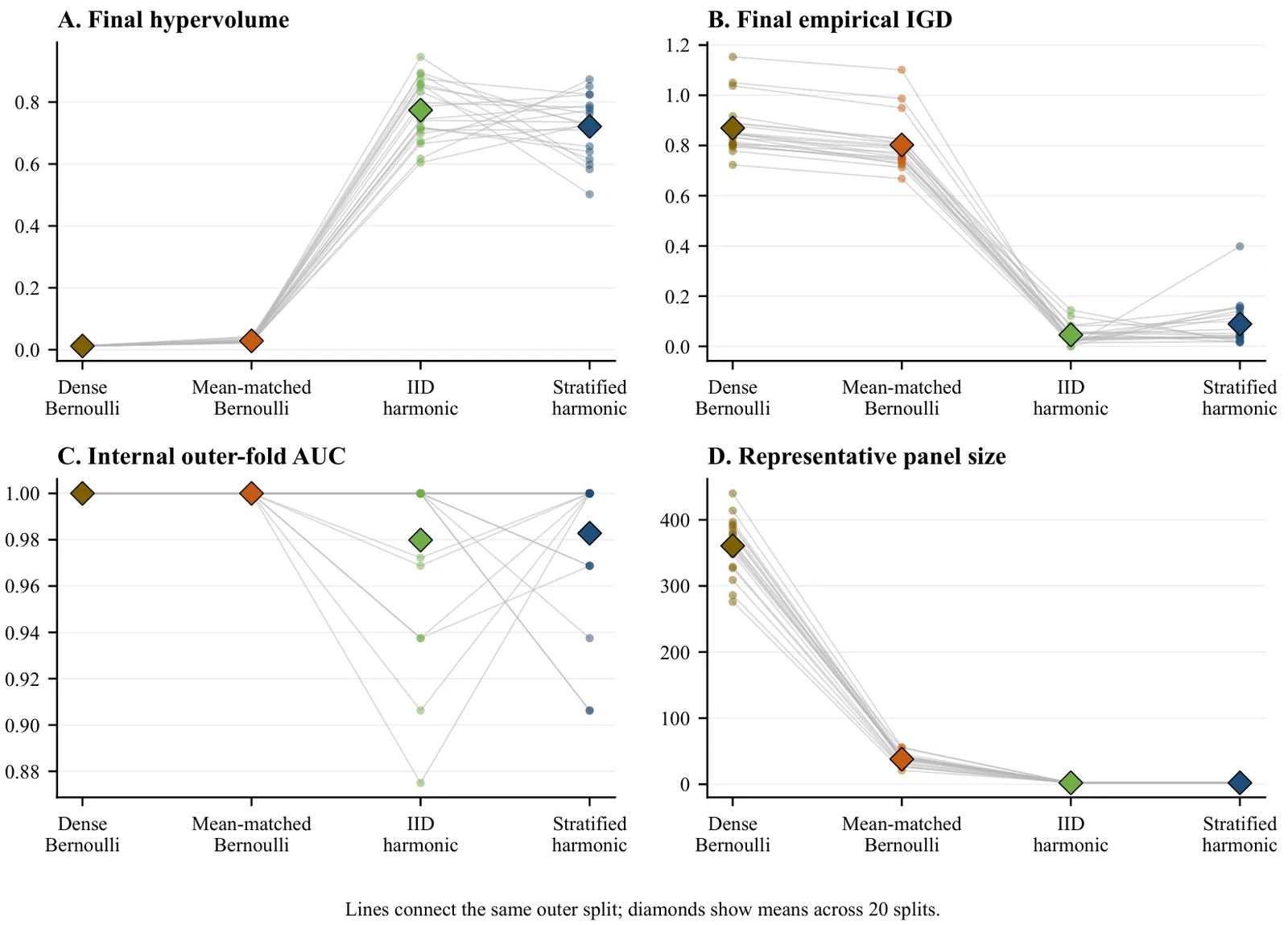
Matched NSGA-II initialization ablation at 10,000 fitness evaluations for the four original conditions. Gray lines connect the same outer split and diamonds show means. HV and empirical IGD use the normalized pooled four-initializer reference within each split; Table 6 gives the seven-condition values. HV, hypervolume; IGD, inverted generational distance.

The initialization ablation separated the harmonic cardinality law, finite-population stratification, and initial density, and it compared harmonic sampling with three existing sparse strategies; see Table 6 and Figure 5. All front descriptors in this subsection use joint normalization across the seven conditions. Stratified and IID harmonic sampling both selected panels averaging 2.15 genes. Their final HV values were 0.736 and 0.787, and their empirical IGD values were 0.109 and 0.086; neither contrast was significant after correction, with $p_{H}=1.000$ for both. Their representative sizes were identical. Stratified quantiles and IID draws from the same harmonic law therefore performed alike.

Expected-size-matched Bernoulli initialization retained panels averaging 38.00 genes, whereas dense Bernoulli initialization retained 360.70. Relative to stratified harmonic sampling, the corrected mean size differences were −35.85 genes ($p_{H}=3.16\times 10^{-6}$) and −358.55 genes ($p_{H}=9.42\times 10^{-12}$). Both Bernoulli controls had much smaller HV and larger IGD ($p_{H}\leq 9.50\times 10^{-10}$ for HV and $p_{H}\leq 2.60\times 10^{-11}$ for IGD). No outer-AUC or outer-balanced-accuracy contrast remained significant.

The three existing sparse strategies reached the compact region as well. The bounded uniform initializer produced panels of 2.30 genes with HV 0.732, close to the 0.736 of stratified harmonic sampling. The SparseEA initializer combined with NSGA-II operators gave more variable results, with panels of 4.65 genes on average, a standard deviation of 4.77 genes, and HV 0.548; its broad initial cardinality distribution left some runs far from the compact region after the 1052 to 1352 scoring evaluations had been spent. The complete SparseEA algorithm, whose score-guided operators keep adding and removing genes during evolution, reached the largest HV, 0.821, and the smallest IGD, 0.051, with panels of 2.05 genes. None of these three contrasts with stratified harmonic sampling was significant after Holm correction for any outcome. The 1/k cardinality law therefore improved finite-budget access to sparse trade-offs relative to Bernoulli initialization, and it performed on a par with existing sparse initializers without requiring a size bound or scoring evaluations. Internal predictive discrimination was the same under every initializer.

### 4.6. GO-Objective Ablation Results

**Figure 6:**
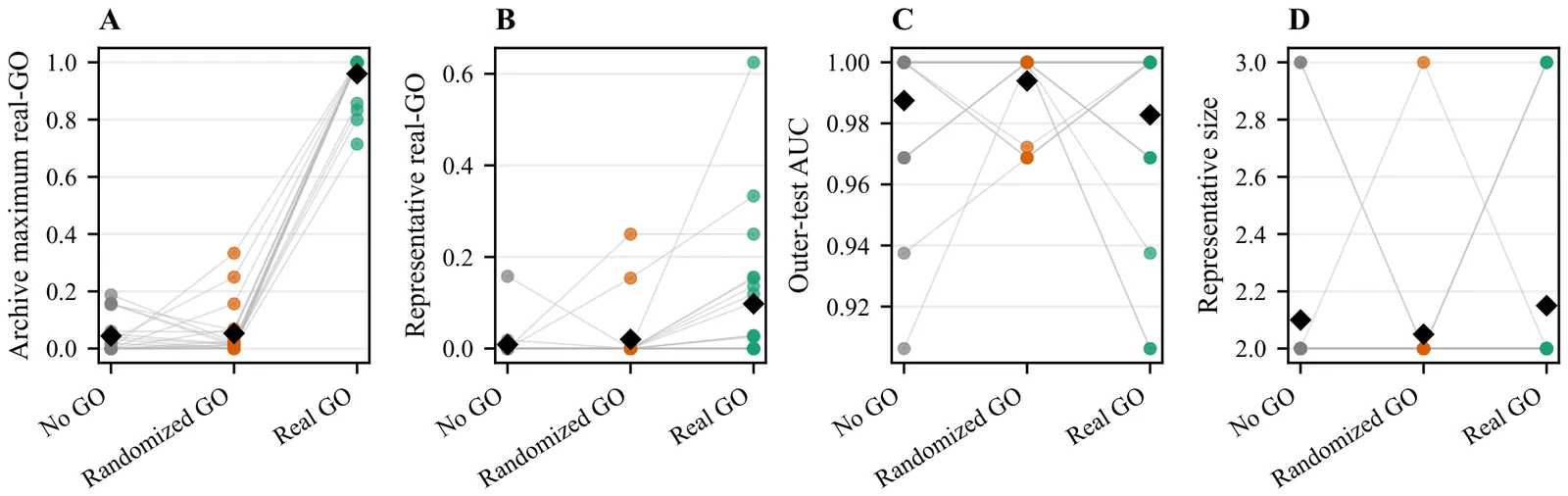
Matched NSGA-II GO-objective ablation. (**A**) Maximum real-GO coherence in the final archive; (**B**) real-GO coherence of the representative panel; (**C**) outer-test AUC; (**D**) representative panel size. Dots show individual splits colored by condition: gray, no GO; orange, randomized GO; green, real GO. Gray lines connect the same outer split; diamonds denote condition means. Real-GO coherence is always calculated using the unpermuted annotation.

The GO objective changed the NSGA-II search archive without improving internal outer-fold discrimination; see Table 7 and Figure 6. Under real GO, the mean maximum real-GO coherence attained anywhere in the final archive was 0.960, compared with 0.044 without GO and 0.053 under randomized GO. The paired mean differences were 0.916 and 0.907, with $p_{H}<4.1\times 10^{-12}$ for both contrasts. Archive means and 90th percentiles showed the same result after corrected inference.

The GO-blind representative panels showed a smaller and more variable change. Their mean real-GO coherence was 0.098 under real GO, 0.009 without GO, and 0.020 under randomized GO; neither corrected contrast was significant ($p_{H}=0.461$). Outer AUC, balanced accuracy, and representative size also did not differ after correction. Thus, real annotation guided the population toward high-coherence regions of the Pareto front, while the accuracy-first decision rule, which ignores GO, carried little of that gain into the single selected panel and left held-out prediction unchanged.

The GO-aware tolerance rule shows how the archive can be used once accuracy has saturated; see Table 8. With δ=0.025, the chosen NSGA-II panels had a mean real-GO coherence of 0.620, compared with 0.098 for the accuracy-first panels, a paired difference of 0.522 with a 95% confidence interval of 0.300 to 0.745 ($p_{H}=0.0006$). NSGA-II tie-break, SPEA2, and SPEA2+ gave the same pattern, with coherence of 0.592 to 0.636. The panels became larger, by 2.10 to 2.60 genes on average, and outer AUC changed by −0.009 to 0.016, with no significant contrast. The two decomposition methods kept fewer high-coherence panels near the accuracy maximum, so the rule raised coherence less, to 0.288 for MOEA/D and 0.198 for MOEA/D-NRD. With δ=0.05 the results were similar. Applied to the no-GO archives, the same rule reached a mean coherence of 0.044, because those archives contained almost no coherent panels; the gain therefore requires optimizing GO during search.

Annotation resources not used during search pointed in the same direction. The largest Reactome coherence in the final NSGA-II archive averaged 0.198 under real GO, 0.036 without GO, and 0.058 under randomized GO, and real GO gave the larger value in 14 and 13 of 20 splits, respectively. For KEGG the archive maxima were 0.306, 0.029, and 0.022 and for MSigDB Hallmark 0.274, 0.106, and 0.122. The split-level values were highly skewed, and the corrected contrasts had $p_{H}\geq 0.390$. Tolerance-rule panels from the four dominance- and strength-based algorithms had Reactome coherence of 0.062 to 0.141, against a random expectation of 0.014 to 0.015, with corrected $p_{H}=1.000$.

### 4.7. Robustness of the Internal Results

Table 9 summarizes the robustness analyses. Across five independent runs per algorithm and split, mean outer AUC was 0.983 to 0.988. The standard deviation of outer AUC between runs on the same split, 0.018 to 0.024, was as large as the standard deviation between splits of the run-averaged values, 0.016 to 0.035. The selected genes changed from run to run: the mean pairwise Jaccard similarity between runs on the same split was 0.010 to 0.122, and no split yielded the same panel in all five runs. With run-averaged values, no pairwise algorithm contrast in AUC, balanced accuracy, or panel size was significant; all six algorithms exceeded random panels, with $p_{H}\leq 0.011$, and none exceeded top-DEG, with $p_{H}=1.000$. Run-averaged hypervolume showed that NSGA-II, NSGA-II tie-break, SPEA2, and SPEA2+ covered more of the front than both MOEA/D variants, with $p_{H}\leq 0.0024$.

Extending outer cross-validation to ten repeats, 50 splits in total, gave mean outer AUC of 0.981 to 0.988. All six algorithms then exceeded random panels after Holm correction, with $p_{H}\leq 0.032$, and the difference from top-DEG stayed between −0.003 and 0.008 with $p_{H}=1.000$ (Supplementary Figure S1). The conclusions drawn from four repeats therefore did not change with more repeats.

Under permuted labels, the FDR screen returned no gene in any of the 100 runs, so the complete pipeline would not have produced a panel. When the 1206 genes with the smallest *p*-values were searched instead, NSGA-II still reached a mean inner-CV balanced accuracy of 0.988, with a minimum of 0.968 across permutations, using 18.58 genes on average. The corresponding outer AUC averaged 0.510, with a standard deviation of 0.096 and a maximum of 0.704 over 20 permutations, compared with 1.000 for the real labels in the same five folds. The held-out outer folds thus behave as expected under the null, whereas the inner fitness after adaptive search carries almost no information about generalization.

Repeating the differential-expression screen inside every inner training fold did not change the results. Inner balanced accuracy remained 1.000 for four algorithms and 0.993 to 0.994 for the MOEA/D variants, outer AUC was 0.964 to 0.994 with no corrected difference from the main analysis, and no algorithm exceeded top-DEG. The selected genes differed from the main analysis, with Jaccard similarity of 0.033 to 0.292, consistent with the instability described above. Removing the GSE30528 coverage restriction did not change internal performance either. The pool then contained 2265 to 2917 passing genes before the 2000-gene cap. NSGA-II reached outer AUC 0.997 against 0.983, a difference of 0.014 (p=0.464), and balanced accuracy 0.934 against 0.894 (p=0.593), with panels of 1.80 genes. However, on average, 49.2% of the genes in each panel were not measured on GSE30528, and 70% of these panels contained at least one such gene.

### 4.8. Feature Recurrence

Representative-panel membership remained unstable across outer training splits. DUSP1 had the largest within-algorithm recurrence, appearing in 6 of 20 SPEA2 panels and 6 of 20 MOEA/D-NRD panels. In NSGA-II, FOS and NFATC3 each appeared in 4 of 20 panels. No gene reached a frequency of 0.5 for any algorithm. Over all 600 runs of the five-run analysis, the most frequent genes were DUSP1 in 100 runs, CNBP in 83, PTOV1 in 75, FOS in 68, NFATC3 in 52, and CHCHD2 in 44.

**Figure 7:**
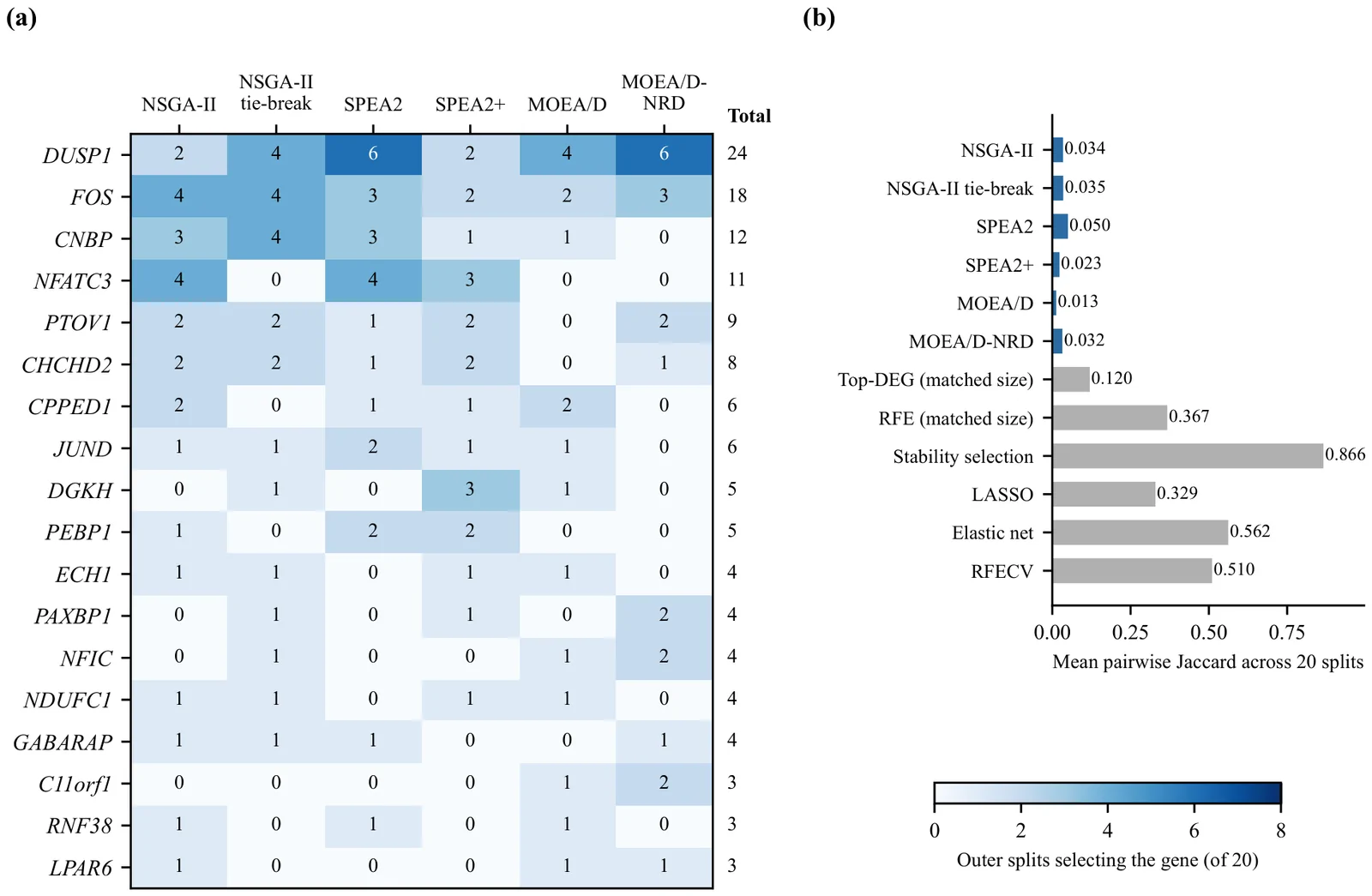
Selection recurrence and stability. (**a**) Recurrence for the 18 genes with the largest total counts across algorithms. Each cell gives the number of outer training splits, from 20, whose real-GO representative panel contained that gene; the right column gives the total over the six algorithms. (**b**) Mean pairwise Jaccard similarity of selected gene sets across the 20 outer splits for the evolutionary algorithms, shown in blue, and for the comparator methods, shown in gray. DEG, differentially expressed gene; RFE, recursive feature elimination; RFECV, RFE with cross-validated size.

Figure 7 shows recurrence for the 18 genes with the largest total counts across algorithms, together with the selection stability of each method. The maximum cells contain DUSP1 in 6 of 20 outer training splits for SPEA2 and MOEA/D-NRD. Counts are generally low and optimizer-dependent, consistent with several interchangeable panels attaining saturated inner fitness.

Although individual genes recurred rarely, the genes selected across splits converged on a few pathways in annotation resources not used for the search. Among the 121 genes that appeared in any real-GO representative panel, tested against the 1686 genes in the union of candidate pools, Reactome Complex I biogenesis contained 6 of its 8 pool genes (FDR 0.002), KEGG oxidative phosphorylation 8 of 26 (FDR 0.025), KEGG diabetic cardiomyopathy 11 of 46 (FDR 0.025), and the Hallmark TNF-α signaling via NF-κB set 12 of 59 (FDR 0.033). The mitochondrial genes were NDUFA2, NDUFAB1, NDUFB5, NDUFB7, NDUFC1, NDUFV1, SDHC, and UQCR10. The TNF-α set consisted mainly of immediate-early and stress-response genes: ATF3, BTG2, DUSP1, FOS, FOSB, JUN, NFIL3, and ZFP36. The oxidative phosphorylation signal persisted in panels found without the GO objective, with Hallmark oxidative phosphorylation at FDR 0.045, so it does not arise from GO guidance. Supplementary Table S9 lists all terms with FDR below 0.05, and Supplementary Data file S10 gives the complete results.

### 4.9. External Cohort-Specific Refitting and Locked Transfer

External feature-set portability was strongly cohort- and algorithm-dependent; see Table 10. In GSE30528, mean AUC ranged from 0.612 for SPEA2+ to 0.726 for NSGA-II and MOEA/D. Only NSGA-II had a positive mean difference from its same-size random-panel average, and that difference was small. In GSE142025, mean AUC ranged from 0.849 for SPEA2+ to 0.964 for MOEA/D-NRD, while balanced accuracy ranged from 0.572 to 0.643. The gap between AUC and thresholded performance arose from the threshold, not from the ranking. At 0.5, refitted models were strongly shifted toward the majority class of each cohort: in GSE30528, with 9 cases among 22 samples, sensitivity was 0.194 to 0.328 and specificity 0.958 to 0.988; in GSE142025, with 27 cases among 36 samples, sensitivity was 0.987 to 0.994 and specificity 0.150 to 0.294. Strong L2 shrinkage with C=0.1 and training folds of 17 to 29 samples pulls predicted probabilities toward the training case proportion, so a fixed 0.5 cut-off assigns most samples to the larger class. When the threshold was set to the case proportion of each training fold, balanced accuracy rose to 0.617 to 0.718 in GSE30528 and to 0.828 to 0.910 in GSE142025 (Table 11).

A total of 4 of the 120 real-GO panels lost one gene in GSE142025 because it was absent from the processed matrix; no panel lost all genes. All genes were available in GSE30528 by design. In the conditional panel-level comparisons, NSGA-II exceeded the top-DEG control in GSE30528 ($p_{H}=0.0079$), and MOEA/D-NRD exceeded its random-panel control in GSE142025 ($p_{H}=0.0238$); no GSE142025 contrast with top-DEG was significant.

Locked transfer was weaker than cohort-specific refitting; see Table 11. With discovery coefficients and the 0.5 threshold applied unchanged, mean AUC across the 20 split models was 0.539 to 0.648 in GSE30528 and 0.702 to 0.791 in GSE142025. Sensitivity was 0.767 to 0.850 and 0.854 to 0.909, and specificity 0.138 to 0.208 and 0.339 to 0.550, so the locked models called most external samples DN. The locked ensembles that averaged the 20 split models reached AUC 0.547 to 0.709 in GSE30528, with all bootstrap intervals including 0.5, and 0.864 to 0.984 in GSE142025, with lower limits of 0.737 to 0.938. Locked evolutionary panels exceeded the same-size top-DEG panels by 0.074 to 0.185 in GSE30528 and 0.092 to 0.214 in GSE142025, but these panel-level differences were not significant after correction, with $p_{H}\geq 0.312$. LASSO, size-matched RFE, and stability-selection panels transferred comparably or better, with locked AUC of 0.639 to 0.700 in GSE30528 and 0.890 to 0.974 in GSE142025.

**Figure 8:**
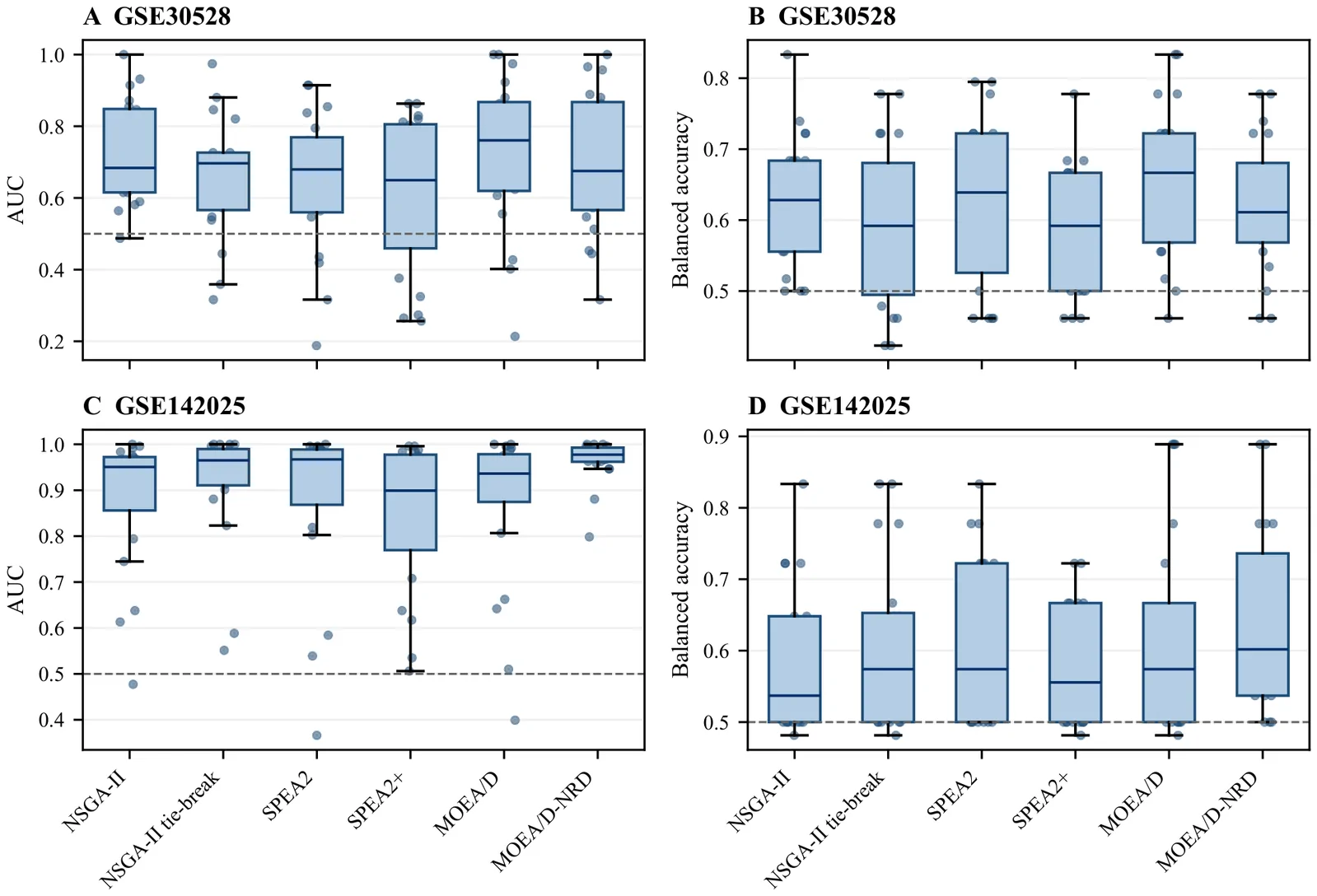
External cohort-specific refitting for 20 fixed real-GO panels per algorithm. Boxes and dots summarize AUC and balanced accuracy; the dashed line marks 0.5. AUC, area under the receiver operating characteristic curve.

Figure 8 shows the complete external distributions. GSE30528 results span a wide range and include AUC values below 0.5 for some panels. GSE142025 generally has higher AUC, while balanced accuracy remains closer to 0.5.

## 5. Discussion

### 5.1. Principal Findings and Contribution

This study contributes a within-run leakage-controlled framework for biologically guided evolutionary gene-panel discovery. Its main value lies in the evaluation protocol and in what that protocol reveals. Its main methodological elements are harmonic sparse initialization over every nonempty cardinality, unrestricted subsequent variation, a three-objective formulation that treats GO coherence as search guidance, and matched no-GO and structure-preserving randomized-GO controls. All supervised discovery steps were repeated within outer training data, while same-size random and top-DEG panels, five established feature-selection methods, repeated search seeds, additional outer repeats, and a permuted-label null separated candidate-pool informativeness from added value due to evolutionary search.

The corrected experiment produced four principal findings. First, compact panels emerged without a cardinality cap: representative sizes averaged 2.10–2.70 genes. Second, the initialization ablation showed that the harmonic cardinality law, and not stratified quantiles alone, enabled finite-budget access to sparse fronts relative to Bernoulli initialization; it performed on a par with the bounded uniform and SparseEA initializers and slightly below the complete SparseEA algorithm. Third, all six algorithms achieved high internal outer-fold AUC, with no corrected predictive difference among them. Four algorithms exceeded random panels in AUC, all six did so with ten outer repeats or with run-averaged values, top-DEG matched them, and LASSO, RFE, and stability selection reached equal or higher discrimination with far more reproducible gene sets. Fourth, real GO annotation strongly redirected the NSGA-II Pareto archive toward functionally coherent panels, and a GO-aware decision rule applied to the same archives raised representative coherence from about 0.1 to about 0.6 with no measurable loss of outer AUC. The GO result demonstrates controllable biological guidance during search.

These results separate improvements in search behavior from improvements in prediction. The harmonic law and the GO objective determine how quickly and where a finite budget reaches compact trade-offs, whereas predictive accuracy and stability are set by the signal in the data and are matched or exceeded by standard embedded selection.

### 5.2. Interpretation of Sparse Panel Emergence and High Internal Discrimination

The search operators could add or remove genes. With per-bit mutation probability 1/m and a sparse chromosome, mutation frequently adds a gene because most bits are zero. Convergence near two genes therefore arose from selection and the representative rule; the operators did not enforce irreversible shrinkage. Inner balanced accuracy reached or approached 1.000 with small panels, after which leanness resolved ties.

Candidate-pool screening used the complete outer training split before the inner folds were formed. Within each final nested run, this preserves the held-out outer fold but can make the inner search fitness optimistic and less discriminating among panels. Adaptive evaluation of approximately 10,000 panels on the same inner folds adds search overfitting. The permuted-label null shows how strong this effect is: with no real signal, the search still reached an inner balanced accuracy of 0.988, while the held-out AUC fell to 0.510. Repeating the differential-expression screen inside the inner folds left the inner fitness and outer performance unchanged, so adaptive search over many panels, and not the screening order, is the main source of inner optimism. The resulting cardinality is best understood as the optimizer’s solution under this training criterion. A fixed-cardinality study would answer a different question about performance at prespecified assay sizes.

The high outer-fold AUC values reflect a strongly separable classification task. DN glomeruli from diagnostic biopsies were compared with glomeruli from the unaffected portions of tumor nephrectomies, and more than a thousand genes passed the differential-expression screen in every split. Random panels drawn from the screened pool already reached outer AUC of about 0.90, top-DEG panels about 0.98, and LASSO 1.000. With four control and eight or nine DN samples per test fold, one misranked pair changes AUC by about 0.03, so the remaining differences among methods are within resampling noise. The external cohorts show lower and more variable values because the platforms, tissue sources, disease stages, and control definitions differ, and because cohort-specific refitting with 17 to 29 training samples adds estimation error. Together with the permuted-label null, the internal estimates show no sign of computational leakage.

The harmonic initializer removes the need to choose an arbitrary maximum panel size while retaining access to large panels. It acts on the initial population and leaves panel size free during evolution. Stratified and IID harmonic sampling produced the same mean representative size and statistically indistinguishable front descriptors. In contrast, both expected-size-matched and dense Bernoulli populations remained much larger after 10,000 evaluations and covered substantially less of the pooled empirical front. This isolates the broad 1/k cardinality law as the supported mechanism: it places some initial solutions directly at small scales while retaining support for large panels. The bounded uniform initializer achieves a similar effect when a sensible upper bound is known, and SparseEA achieves it through scoring evaluations and score-guided operators. The harmonic law needs neither, which makes it a convenient default for binary MOEAs; in this problem, the complete SparseEA algorithm covered the front slightly better. Outer-fold discrimination was the same under every initializer.

### 5.3. Algorithm Comparison and Decision Rules

No single algorithm led every outcome. SPEA2+ had the best numerical front descriptors, whereas MOEA/D had the largest internal outer-fold AUC, MOEA/D-NRD had the largest balanced accuracy and the strongest GSE142025 portability, and NSGA-II tied MOEA/D for the highest GSE30528 AUC. Corrected predictive comparisons were null, and they stayed null when each split was represented by the average of five independent runs. Run-to-run variation on the same split was as large as variation between splits, so ranking algorithms requires several runs per split. Exploratory development informed the designation of NSGA-II as the final GO-ablation reference; the corrected six-algorithm outcomes were not used to revise that choice.

The accuracy-first, GO-blind representative rule deliberately favors predictive fitness over biological coherence. This explains why real GO had a large archive-level effect but a smaller representative-panel effect. Applications that require a biologically coherent final panel should prespecify a decision rule that uses all three objectives, such as a knee point, a minimum acceptable predictive fitness followed by GO coherence, or a clinically defined assay-size range. The tolerance rule reported here illustrates this approach. It shows that, once inner accuracy has saturated, the archive contains panels about two genes larger, whose members share GO processes, and that choosing them costs no measurable outer AUC.

The random and top-DEG controls lead to complementary conclusions. Improvement over random subsets shows that several algorithms exploited informative structure within the screened candidate pool. Parity with top-DEG shows that a strong, inexpensive marginal-ranking baseline already captures the predictive signal in this cohort. The embedded and wrapper baselines confirm this. LASSO and stability selection reached perfect outer AUC with one to five genes and selected the same genes far more often across splits, with mean Jaccard similarity of 0.329 and 0.866 against at most 0.050 for the evolutionary panels. When the inner fitness saturates, many two-gene panels are equally fit, and a stochastic search returns a different one each time; a penalized model breaks these ties consistently through its coefficient path. The multi-objective archives additionally expose explicit trade-offs and permit biological steering, which a single ranked list does not provide.

For a practitioner, the framework is therefore useful as an exploratory and auditing tool. Its archives show which panel sizes and levels of functional coherence are attainable at a given internal accuracy, the GO objective can steer the choice toward interpretable panels without a measurable loss of discrimination, and the nested design with matched random, top-DEG, and permuted-label controls shows how much of the apparent performance is due to search itself. When the aim is a single reproducible predictive panel from a cohort of this size, regularized regression or stability selection is the simpler and more stable choice.

### 5.4. Meaning of the GO Objective

The GO objective was defined as the mean pairwise Jaccard similarity of retained Biological Process memberships. Unlike a nearest-neighbor maximum, its random expectation does not increase mechanically with panel cardinality. The randomized-GO condition preserved annotation counts, term sizes, and the complete similarity distribution while breaking gene-to-annotation identity. The large archive-level difference between real and randomized GO therefore shows that the optimizer responded to biologically matched annotation structure, beyond the generic effect of adding a third numerical objective.

This evidence is target attainment: real GO guides the population toward a different region of the search space. Reactome, KEGG, and Hallmark coherence, which were not optimized, was higher in real-GO archives in most splits, so GO guidance carries over in part to other curated resources.

Held-out prediction was unchanged by coherence for three reasons. First, inner accuracy was saturated, so any gain would have had to appear as a difference among panels that already classify the training data perfectly. Second, functional coherence and discriminative power are different properties: genes that share GO processes tend to be co-expressed and carry redundant information, whereas the best two-gene discriminators combine complementary signals. Third, the accuracy-first representative rule ignored the GO objective by design. Coherence is thus a criterion for interpretability and assay design, best judged by functional experiments.

### 5.5. Biological Interpretation of the Selected Genes

Individual genes recurred rarely, but the selected genes converged on two programs. The first is mitochondrial oxidative phosphorylation, represented by complex I subunits such as NDUFA2, NDUFB5, and NDUFC1, the succinate dehydrogenase subunit SDHC, and UQCR10. This signal persisted without the GO objective. Mitochondrial dysfunction links hyperglycemia to excess superoxide production and oxidative stress in diabetic complications [7,8], and the selection of SOD1 in several runs points to cellular antioxidant defense, to which selenoproteins also contribute [10]. The KEGG diabetic cardiomyopathy term was enriched through these same mitochondrial genes, which indicates shared metabolic injury across diabetic complications. Tissue-specific regulation nevertheless differs between organs: in diabetic cardiomyopathy, for example, the circular RNA DICAR regulates cardiomyocyte pyroptosis [59]. Reviews of antidiabetic mechanisms likewise describe inflammation, oxidative stress, and metabolic signaling as interacting pathways [60]; that literature concerns therapeutic agents, whereas the present analysis describes tissue expression differences.

The second program consists of immediate-early and stress-response genes, including FOS, FOSB, JUN, ATF3, DUSP1, ZFP36, and NFIL3, which belong to the Hallmark TNF-α signaling via NF-κB set. These genes respond to inflammatory and innate immune signaling, which is active in the diabetic kidney [9], and DUSP1, the most frequently selected gene, attenuated renal injury in an experimental DN model [61]. ATF3 is a stress-inducible transcription factor, and GABARAP and GABARAPL1 take part in autophagy; endoplasmic-reticulum stress, the unfolded protein response, and autophagy are all implicated in diabetic nephropathy [62]. NFATC3 belongs to the calcineurin–NFAT pathway, whose activation in podocytes causes glomerulosclerosis [63]. Immediate-early genes, including JUND and ATF3, are also induced within one hour of warm ischemia in surgical specimens [64]. Because the controls in GSE96804 and GSE142025 came from tumor nephrectomies, whereas the cases included diagnostic biopsies, part of this signal may reflect procurement differences.

Extracellular-matrix and vascular programs, including endothelial-to-mesenchymal transition and its RNA-mediated regulation, contribute to diabetic kidney injury [65]. Such genes were present in the candidate pools but were rarely chosen, because two-gene panels from the mitochondrial and stress-response programs already separated the training samples perfectly. The gene-level findings are hypotheses for targeted validation, and the recurrence data point to several interchangeable signatures.

### 5.6. Interpretation of External Cohorts

Two transfer analyses were performed. In cohort-specific refitting, only gene identities were transferred to the external cohorts. Each scaler and logistic-regression model was refitted inside that cohort’s cross-validation, so the analysis evaluates feature-set portability. GSE30528 produced moderate and variable discrimination; GSE142025 produced higher AUC for several algorithms, especially MOEA/D-NRD, but thresholded balanced accuracy was lower. The low balanced accuracy arose mainly from the fixed 0.5 threshold. Strongly regularized models fitted to small training folds return probabilities close to the training case proportion, so most samples fall into the majority class, and a threshold matched to the training case proportion raised balanced accuracy to 0.617 to 0.718 and 0.828 to 0.910. In locked transfer, discovery coefficients and the 0.5 threshold were applied unchanged after within-cohort standardization. Discrimination was lower than with refitting, and the locked models assigned most external samples to DN. The locked evolutionary panels discriminated better than locked top-DEG panels without a corrected difference, and locked LASSO, RFE, and stability-selection panels transferred comparably or better. Within-cohort standardization removes platform scale differences and assumes a similar case mix across cohorts, which explains the shifted thresholds. Cohort-dependent rankings reinforce the absence of a universal algorithm winner.

The cohorts compare DN with non-DN kidney tissue. GSE96804 and GSE30528 contain microdissected glomerular profiles. GSE142025 contains kidney-biopsy and non-DN nephrectomy tissue, with early and advanced DN represented among cases. Tissue composition, fibrosis, inflammation, procurement, assay platform, disease stage, and center therefore contribute to discrimination, and the panels capture differences between diseased and non-diseased kidney tissue. The cohorts also differ in population. GSE96804 and GSE142025 were collected in Chinese centers and GSE30528 in an ethnically diverse North American population, and in GSE30528, the only cohort with reported age, cases were older than controls. Comorbidity, aging, and treatment influence DKD risk and progression [66], and ethnicity, socioeconomic conditions, and environmental exposures differ between cohorts.

### 5.7. Limitations and Future Work

GSE96804 supported exploratory method development before the final protocol was locked, so the outer-fold values are internal resampling estimates of a protocol developed on the same data. The discovery cohort is small, the repeated splits reuse its 61 patients, the corrected inference is approximate, and the external cohorts contain 22 and 36 samples, so external P-values describe consistency within these cohorts. The GSE30528 platform restricted assay availability; without the restriction, internal performance was unchanged but about half of the genes in each panel could not have been measured in GSE30528. The cohorts compare DN with non-DN kidney tissue without sample-level clinical data, so the panels are not a clinically validated biomarker, and their separation of DN from other kidney diseases and from diabetic kidneys without nephropathy remains to be tested. The fixed logistic-regression hyperparameter and the accuracy-first representative rule are additional design choices.

Future work should test the framework in larger cohorts in which inner accuracy does not saturate, compare decision rules prespecified before analysis, and combine the GO objective with stability-oriented objectives so that the search favors reproducible panels. Biological follow-up should use an independent annotation source or experimental assay. Clinical validation should transfer one locked preprocessing–panel–classifier pipeline to adequately powered cohorts containing diabetic patients with and without nephropathy and relevant alternative kidney diseases.

## 6. Conclusions

This study establishes a reproducible evaluation framework for sparse, biologically guided evolutionary gene-panel discovery in small transcriptomic cohorts. Its contributions are methodological and concern search behavior and evaluation. Harmonic sparse initialization allowed every nonempty panel size while avoiding a fixed cardinality cap. Stratified and IID harmonic variants were comparable, whereas expected-size-matched and dense Bernoulli controls retained much larger panels and poorer final fronts at the same budget. This supports the harmonic cardinality law as a finite-budget search mechanism, and its fronts were comparable to those of existing sparse initializers without requiring a size bound or scoring evaluations. Across six algorithms, the framework produced high internal outer-fold discrimination and explicit three-objective Pareto fronts; top-DEG matched their discrimination, and regularized regression and stability selection matched it with much more reproducible genes. Repeated search runs, additional outer repeats, inner-fold screening, and a permuted-label null confirmed these results and showed that the held-out outer folds, and not the inner fitness after adaptive search, measure generalization.

The GO ablation clarifies the role of biological guidance: real annotation strongly redirected the nondominated archive toward high GO coherence compared with both removing GO and randomizing gene–annotation identity, and a GO-aware decision rule turned this archive-level effect into coherent representative panels at the same outer AUC. The selected genes pointed to mitochondrial oxidative phosphorylation and immediate-early stress signaling and provide hypotheses for targeted validation. These findings provide a clear basis for developing prespecified multi-objective decision rules, adding stability-oriented objectives, and validating locked panels in larger clinically representative cohorts.

## Figures and Tables

**Table 1:** Cohorts, sample characteristics available in the public records, and analytical roles. Counts are DN/control.

GEO ID	Assay and Tissue	Samples	Cases and Controls	Role
GSE96804	Affymetrix HTA 2.0; microdissected glomeruli	41/20	Type 2 diabetes with biopsy-confirmed DN; controls from the unaffected portion of tumor nephrectomies with eGFR above 90 mL/min and no proteinuria [3]. Men 29 of 41 cases and 14 of 20 controls.	Nested differential-expression screening, evolutionary search, and outer testing [3].
GSE30528	Affymetrix HG-U133A 2.0; microdissected glomeruli	9/13	DKD from diagnostic biopsies or nephrectomies; controls from living donors or nephrectomies. Women 5 of 9 cases and 5 of 13 controls; mean age 64.0 and 51.4 years [1].	Cohort-specific panel refitting and locked transfer; gene coverage also defined assay availability [1].
GSE142025	Illumina HiSeq 4000 RNA-seq; kidney biopsy or non-DN nephrectomy tissue	27/9	Biopsy-proven DN, 6 early and 21 advanced; controls from the unaffected portion of tumor nephrectomies. Sex not reported per sample.	Cross-platform cohort-specific panel refitting and locked transfer [2].

The GSE142025 report described 28 DN and 9 control samples; the processed expression matrix and matching metadata contained 27 DN and 9 control profiles. All three cohorts compare DN kidney tissue with non-DN kidney tissue. Age, diabetes duration, glycated hemoglobin, estimated glomerular filtration rate, proteinuria, and treatment were not available at sample level in the GEO records, so they could not be used for adjustment or stratification.

**Table 2:** Optimization parameters for the six multi-objective evolutionary algorithms.

Algorithm	Search Structure	Size	Algorithm-Specific Configuration
NSGA-II	Dominance and crowding	100	Standard rank-and-crowding survival.
NSGA-II tie-break	Dominance and crowding	100	Balanced tie-break in the critical front.
SPEA2	Strength and archive	100	Standard strength, density, and archive update.
SPEA2+	Strength and archive	100	Normalized distance-chain archive completion.
MOEA/D	Decomposition	105	15 neighbors; mating probability 0.7.
MOEA/D-NRD	Decomposition	105	Neighborhood-region replacement; 15 neighbors.

Size denotes population size for the NSGA-II and SPEA2 families and reference-direction subproblems for the MOEA/D family. Common settings were a split-specific binary vector of length *m*, harmonic sparse initialization, a 10,000-evaluation target, crossover probability 0.9, mutation probability 1/m, and no cardinality repair.

**Table 3:** Primary internal outer-fold results across 20 matched splits. Values are the mean (SD). Sens; Spec gives sensitivity and specificity at threshold 0.5 after averaging each sample’s four out-of-fold probabilities. Differences compare each evolutionary panel with its split-matched control. Each difference cell reports ΔAUC, its 95% confidence interval, and Holm-adjusted $p_{H}$ from the Nadeau–Bengio corrected resampled *t* approximation; adjustment was across six algorithms within each control. AUC, area under the receiver operating characteristic curve; BA, balanced accuracy; DEG, differentially expressed gene; SD, standard deviation.

Algorithm	Size	Outer AUC	Outer BA	Sens; Spec	vs. Random ΔAUC [95% CI]; $p_{H}$	vs. Top-DEG ΔAUC [95% CI]; $p_{H}$
NSGA-II	2.15 (0.37)	0.983 (0.031)	0.894 (0.091)	1.000; 0.850	0.081 [0.032, 0.129]; 0.0158	0.005 [−0.049, 0.058]; 1.000
NSGA-II tie-break	2.20 (0.41)	0.981 (0.028)	0.907 (0.087)	1.000; 0.950	0.077 [0.021, 0.133]; 0.0403	0.002 [−0.039, 0.043]; 1.000
SPEA2	2.15 (0.37)	0.973 (0.058)	0.912 (0.098)	1.000; 0.950	0.070 [−0.001, 0.141]; 0.1050	0.001 [−0.085, 0.088]; 1.000
SPEA2+	2.10 (0.31)	0.984 (0.031)	0.922 (0.086)	1.000; 0.900	0.083 [0.028, 0.138]; 0.0253	0.012 [−0.048, 0.073]; 1.000
MOEA/D	2.55 (0.60)	0.991 (0.029)	0.900 (0.119)	1.000; 1.000	0.073 [0.019, 0.126]; 0.0403	0.009 [−0.043, 0.060]; 1.000
MOEA/D-NRD	2.70 (0.57)	0.987 (0.039)	0.932 (0.096)	1.000; 1.000	0.061 [−0.003, 0.124]; 0.1050	0.007 [−0.027, 0.040]; 1.000

**Table 4:** Evolutionary panels and established feature-selection methods on the same 20 outer splits. Size, outer AUC, and outer balanced accuracy are means. Selected gene sets were scored with the same standardized L2 logistic-regression classifier. Jaccard is the mean pairwise Jaccard similarity of selected sets across splits; Nogueira is the stability index of Nogueira et al.; maximum frequency is the largest proportion of splits selecting one gene. AUC, area under the receiver operating characteristic curve; BA, balanced accuracy; DEG, differentially expressed gene; RFE, recursive feature elimination; RFECV, RFE with cross-validated size.

Method	Size	Outer AUC	Outer BA	Jaccard	Nogueira	Max. Frequency
NSGA-II	2.15	0.983	0.894	0.034	0.048	0.20
NSGA-II tie-break	2.20	0.981	0.907	0.035	0.047	0.20
SPEA2	2.15	0.973	0.912	0.050	0.072	0.30
SPEA2+	2.10	0.984	0.922	0.023	0.029	0.15
MOEA/D	2.55	0.991	0.900	0.013	0.017	0.20
MOEA/D-NRD	2.70	0.987	0.932	0.032	0.043	0.30
Top-DEG, NSGA-II size	2.15	0.978	0.869	0.120	0.170	0.50
LASSO	4.85	1.000	0.950	0.329	0.475	0.90
Elastic net	227.80	1.000	1.000	0.562	0.681	1.00
RFE, NSGA-II size	2.15	0.998	0.869	0.367	0.462	0.80
RFECV	124.00	1.000	1.000	0.510	0.647	1.00
Stability selection	1.15	1.000	0.838	0.866	0.883	1.00

**Table 5:** Three-objective front descriptors across 20 outer training splits. Values are the mean (SD). Larger HV and smaller IGD indicate better front coverage. Indented rows give Holm-adjusted $p_{H}$ versus MOEA/D-NRD from the Nadeau–Bengio corrected resampled *t* approximation; adjustment was across all 15 algorithm pairs for each indicator. HV, hypervolume; IGD, inverted generational distance; SD, standard deviation.

Algorithm	Hypervolume	Empirical IGD	Archive Size
NSGA-II	0.620 (0.096)	0.110 (0.119)	17.30
$p_{H}$ vs. MOEA/D-NRD	0.0374	0.1376	–
NSGA-II tie-break	0.646 (0.105)	0.096 (0.105)	15.00
$p_{H}$ vs. MOEA/D-NRD	0.0212	0.0783	–
SPEA2	0.625 (0.077)	0.114 (0.105)	16.60
$p_{H}$ vs. MOEA/D-NRD	0.0056	0.1089	–
SPEA2+	0.655 (0.089)	0.088 (0.083)	17.05
$p_{H}$ vs. MOEA/D-NRD	0.0118	0.2281	–
MOEA/D	0.392 (0.126)	0.304 (0.130)	77.10
$p_{H}$ vs. MOEA/D-NRD	1.000	1.000	–
MOEA/D-NRD (reference)	0.294 (0.140)	0.366 (0.153)	79.05

**Table 6:** NSGA-II initialization ablation across 20 matched outer splits, with the complete SparseEA algorithm added for comparison. Values are the mean (SD). All conditions used 10,000-evaluation budgets; for the two SparseEA conditions, the single-gene scoring evaluations were counted within this budget. HV and IGD use a common seven-condition, split-specific normalized reference. Indented rows report Holm-adjusted $p_{H}$ versus stratified harmonic initialization from the Nadeau–Bengio corrected resampled *t* approximation, with adjustment across six controls for each outcome. AUC, area under the receiver operating characteristic curve; BA, balanced accuracy; HV, hypervolume; IGD, inverted generational distance; SD, standard deviation.

Initializer	Size	Outer AUC	Outer BA	HV	Empirical IGD
Stratified harmonic (reference)	2.15 (0.37)	0.983 (0.031)	0.894 (0.091)	0.736 (0.106)	0.109 (0.082)
IID harmonic	2.15 (0.37)	0.980 (0.037)	0.913 (0.096)	0.787 (0.098)	0.086 (0.067)
$p_{H}$	1.000	1.000	1.000	1.000	1.000
Bounded uniform, 1–50	2.30 (0.47)	0.981 (0.041)	0.878 (0.100)	0.732 (0.122)	0.114 (0.085)
$p_{H}$	1.000	1.000	1.000	1.000	1.000
SparseEA initializer	4.65 (4.77)	0.992 (0.028)	0.928 (0.084)	0.548 (0.323)	0.275 (0.242)
$p_{H}$	1.000	1.000	1.000	1.000	0.9920
SparseEA, complete	2.05 (0.22)	0.977 (0.054)	0.901 (0.104)	0.821 (0.086)	0.051 (0.043)
$p_{H}$	1.000	1.000	1.000	1.000	0.9920
Mean-matched Bernoulli	38.00 (8.92)	1.000 (0.000)	0.997 (0.012)	0.028 (0.006)	0.792 (0.104)
$p_{H}$	$3.16\times 10^{-6}$	1.000	0.3078	$9.50\times 10^{-10}$	$2.60\times 10^{-11}$
Dense Bernoulli	360.70 (40.64)	1.000 (0.000)	1.000 (0.000)	0.012 (0.000)	0.857 (0.099)
$p_{H}$	$9.42\times 10^{-12}$	1.000	0.2767	$7.67\times 10^{-10}$	$3.11\times 10^{-12}$

**Table 7:** NSGA-II GO-objective ablation across 20 matched outer splits. Values are the means; predictive metrics and representative GO coherence refer to the GO-blind representative, whereas archive maximum describes target attainment anywhere on the final nondominated front. Indented rows report Holm-adjusted $p_{H}$ versus real GO from the Nadeau–Bengio corrected resampled *t* approximation, with adjustment across the two contrasts for each outcome. AUC, area under the receiver operating characteristic curve; BA, balanced accuracy; GO, Gene Ontology.

Condition	Size	Outer AUC	Outer BA	Representative GO	Archive Maximum GO
Real GO (reference)	2.15	0.983	0.894	0.098	0.960
No GO	2.10	0.988	0.922	0.009	0.044
$p_{H}$	1.000	1.000	1.000	0.461	$3.57\times 10^{-12}$
Randomized GO	2.05	0.994	0.897	0.020	0.053
$p_{H}$	1.000	1.000	1.000	0.461	$4.06\times 10^{-12}$

**Table 8:** Accuracy-first and GO-aware representative panels from the same real-GO archives. Each pair of values gives the accuracy-first panel and the tolerance-rule panel with δ=0.025, averaged over 20 outer splits. $p_{H}$ refers to the paired difference, Holm-adjusted across six algorithms. The last column gives the Reactome coherence of the tolerance-rule panel and the mean coherence of 200 random same-size panels from the candidate pool. AUC, area under the receiver operating characteristic curve; BA, balanced accuracy.

Algorithm	Size	Real-GO	$p_{H}$	Outer AUC	$p_{H}$	Outer BA	Reactome; Random
NSGA-II	2.15/4.40	0.098/0.620	0.0006	0.983/0.975	1.000	0.894/0.884	0.072; 0.015
NSGA-II tie-break	2.20/4.30	0.119/0.618	0.0006	0.981/0.980	1.000	0.907/0.894	0.070; 0.015
SPEA2	2.15/4.75	0.119/0.592	0.0006	0.973/0.989	1.000	0.912/0.934	0.062; 0.014
SPEA2+	2.10/4.50	0.127/0.636	0.0006	0.984/0.975	1.000	0.922/0.916	0.141; 0.014
MOEA/D	2.55/4.40	0.094/0.288	0.0496	0.991/0.988	1.000	0.900/0.922	0.151; 0.015
MOEA/D-NRD	2.70/3.20	0.156/0.198	0.2203	0.987/0.989	1.000	0.932/0.944	0.042; 0.014

**Table 9:** Robustness of internal outer-fold results. Five runs: mean outer AUC across five independent search runs on each of 20 splits, standard deviation of AUC between runs within a split and between run-averaged splits, mean pairwise Jaccard similarity of panels between runs on the same split, and Holm-adjusted $p_{H}$ versus top-DEG for run-averaged values. Ten repeats: mean outer AUC across 50 splits, and ΔAUC with $p_{H}$ versus top-DEG. Inner screen: mean outer AUC when differential-expression screening was repeated inside each inner fold, with $p_{H}$ versus the main analysis. AUC, area under the receiver operating characteristic curve; DEG, differentially expressed gene; SD, standard deviation.

Algorithm	Five Runs per Split					Ten Repeats		Inner Screen
	AUC	SD Within	SD Between	Jaccard	$p_{H}$ Top-DEG	AUC	Δ Top-DEG; $p_{H}$	AUC; $p_{H}$
NSGA-II	0.986	0.021	0.023	0.091	1.000	0.985	0.003; 1.000	0.988; 1.000
NSGA-II tie-break	0.985	0.020	0.028	0.100	1.000	0.983	−0.003; 1.000	0.986; 1.000
SPEA2	0.983	0.018	0.035	0.106	1.000	0.981	0.003; 1.000	0.983; 1.000
SPEA2+	0.987	0.024	0.016	0.122	1.000	0.987	0.008; 1.000	0.964; 1.000
MOEA/D	0.987	0.019	0.019	0.015	1.000	0.986	−0.001; 1.000	0.994; 1.000
MOEA/D-NRD	0.988	0.018	0.017	0.010	1.000	0.988	0.005; 1.000	0.983; 1.000

**Table 10:** External feature-set portability under cohort-specific five-fold refitting. Values are the mean (SD) across 20 fixed panels. Control cells report ΔAUC and Holm-adjusted $p_{H}$ from the Nadeau–Bengio corrected resampled *t* approximation, with adjustment across six algorithms within each cohort and control. AUC, area under the receiver operating characteristic curve; BA, balanced accuracy; DEG, differentially expressed gene; SD, standard deviation.

Cohort	Algorithm	AUC	BA	vs. Random ΔAUC; $p_{H}$	vs. Top-DEG ΔAUC; $p_{H}$
GSE30528	NSGA-II	0.726 (0.143)	0.628 (0.093)	0.027; 1.000	0.276; 0.0079
	NSGA-II tie-break	0.657 (0.165)	0.598 (0.115)	−0.044; 1.000	0.201; 0.4865
	SPEA2	0.650 (0.194)	0.621 (0.117)	−0.050; 1.000	0.195; 0.4865
	SPEA2+	0.612 (0.213)	0.582 (0.092)	−0.086; 1.000	0.155; 0.4865
	MOEA/D	0.726 (0.209)	0.658 (0.108)	−0.005; 1.000	0.272; 0.4154
	MOEA/D-NRD	0.694 (0.191)	0.625 (0.096)	−0.048; 1.000	0.221; 0.4865
GSE142025	NSGA-II	0.880 (0.149)	0.580 (0.103)	0.048; 1.000	0.115; 1.000
	NSGA-II tie-break	0.914 (0.127)	0.606 (0.118)	0.080; 1.000	0.148; 1.000
	SPEA2	0.881 (0.179)	0.616 (0.117)	0.049; 1.000	0.103; 1.000
	SPEA2+	0.849 (0.162)	0.572 (0.083)	0.024; 1.000	0.084; 1.000
	MOEA/D	0.868 (0.175)	0.618 (0.141)	0.032; 1.000	0.081; 1.000
	MOEA/D-NRD	0.964 (0.048)	0.643 (0.129)	0.109; 0.0238	0.171; 0.3398

**Table 11:** Threshold-based external results and locked-model transfer. Refit columns use cohort-specific five-fold refitting: AUC, sensitivity and specificity at 0.5, and balanced accuracy at a threshold equal to the training-fold case proportion. Locked columns apply the GSE96804 split models unchanged after label-free within-cohort standardization: mean AUC and mean sensitivity and specificity at 0.5 across 20 split models, and AUC of the locked ensemble with a stratified bootstrap 95% interval. Top-DEG panels in this table and in the locked comparisons consisted of the first |S| genes of the discovery ranking, from which genes absent from the external cohort were then removed; in GSE142025 they therefore differed by one gene from the effective panel size in at most three of 20 splits, whereas the top-DEG controls in Table 10 were drawn from the available genes at the effective panel size. AUC, area under the receiver operating characteristic curve; BA, balanced accuracy; DEG, differentially expressed gene; RFE, recursive feature elimination.

Cohort	Panel	Cohort-Specific Refitting			Locked Transfer		
		AUC	Sens; Spec	BA, Prevalence	AUC	Sens; Spec	Ensemble AUC [95% CI]
GSE30528	NSGA-II	0.726	0.294; 0.962	0.718	0.594	0.817; 0.188	0.590 [0.325, 0.838]
	NSGA-II tie-break	0.657	0.239; 0.958	0.677	0.585	0.767; 0.185	0.573 [0.316, 0.821]
	SPEA2	0.650	0.283; 0.958	0.659	0.648	0.828; 0.208	0.675 [0.419, 0.906]
	SPEA2+	0.612	0.194; 0.969	0.617	0.539	0.817; 0.138	0.547 [0.299, 0.778]
	MOEA/D	0.726	0.328; 0.988	0.716	0.647	0.839; 0.200	0.709 [0.453, 0.897]
	MOEA/D-NRD	0.694	0.278; 0.973	0.683	0.632	0.850; 0.192	0.650 [0.402, 0.863]
	Top-DEG, NSGA-II size	0.449	0.067; 0.962	0.507	0.466	0.744; 0.108	0.368 [0.137, 0.607]
	LASSO	0.782	0.411; 0.942	0.733	0.639	0.867; 0.258	0.650 [0.402, 0.872]
	RFE, NSGA-II size	0.720	0.328; 0.935	0.694	0.700	0.839; 0.300	0.735 [0.504, 0.932]
	Stability selection	0.592	0.111; 0.915	0.604	0.691	0.883; 0.319	0.684 [0.427, 0.897]
GSE142025	NSGA-II	0.880	0.987; 0.172	0.844	0.741	0.857; 0.389	0.881 [0.757, 0.979]
	NSGA-II tie-break	0.914	0.989; 0.222	0.872	0.770	0.909; 0.444	0.959 [0.889, 1.000]
	SPEA2	0.881	0.993; 0.239	0.847	0.746	0.881; 0.467	0.909 [0.798, 0.992]
	SPEA2+	0.849	0.994; 0.150	0.828	0.702	0.854; 0.339	0.864 [0.737, 0.975]
	MOEA/D	0.868	0.991; 0.244	0.840	0.784	0.878; 0.478	0.951 [0.872, 1.000]
	MOEA/D-NRD	0.964	0.991; 0.294	0.910	0.791	0.893; 0.550	0.984 [0.938, 1.000]
	Top-DEG, NSGA-II size	0.767	0.994; 0.006	0.756	0.600	0.819; 0.194	0.626 [0.453, 0.811]
	LASSO	0.991	0.978; 0.794	0.974	0.923	0.922; 0.656	0.967 [0.905, 1.000]
	RFE, NSGA-II size	0.949	0.978; 0.589	0.936	0.890	0.935; 0.767	0.996 [0.975, 1.000]
	Stability selection	0.970	0.963; 0.272	0.981	0.974	0.963; 0.906	0.971 [0.901, 1.000]

## Data Availability

The data are available in the NCBI Gene Expression Omnibus (GSE96804, GSE30528, GSE142025). The source code is openly available at https://github.com/Yilei-11/Diabetic-Nephropathy (accessed on 14 October 2026). Split-level results of all analyses are provided in the Supplementary Data file.

## Supplementary Materials

The following supporting information can be downloaded at https://www.mdpi.com/article/10.3390/bioengineering13101229/s1, Supplementary_Material.pdf: Section S1: Experimental matrix and retained outputs; Section S2: Corrected algorithm comparisons; Section S3: Search-front comparisons; Section S4: GO-objective ablation; Section S5: Feature recurrence; Section S6: External feature-set portability; Section S7: Initialization ablation; Section S8: Reproducibility details; Section S9: Repeated search runs; Section S10: Comparator feature-selection methods; Section S11: Pathway over-representation of selected genes; Section S12: GO-aware representative rules and independent annotation; Section S13: Robustness of internal estimates; Table S1: Complete corrected pairwise comparisons of representative-panel outcomes; Table S2: Complete corrected pairwise comparisons of three-objective front descriptors; Table S3: Corrected NSGA-II GO-ablation contrasts; Table S4: Genes recurring in at least two real-GO representative panels for an algorithm; Table S5: External cohort-specific refitting summaries for the real-GO representative panels; Table S6: Corrected NSGA-II initialization-ablation contrasts; Table S7: Five independent search runs per algorithm and outer split; Table S8: Paired differences between evolutionary panels and established feature-selection methods on the same 20 outer splits; Table S9: Over-representation of selected genes in Reactome 2022, KEGG 2021 Human, and MSigDB Hallmark 2020 against the union of the 20 candidate pools, 1686 genes; Table S10: Representative rules applied to the final archives and coherence under annotation resources not used during search; Figure S1: Robustness of internal estimates. Supplementary_Data.zip: Split-level results of all analyses, files S1–S12.
