# Supplementary material for: Nested Evaluation of Sparse Multi-Objective Evolutionary Gene-Panel Discovery for Diabetic Nephropathy
Source: Bioengineering (Basel). 2026 Oct 21;13(10):1229. doi: 10.3390/bioengineering13101229 (PMC13701386; doi:10.3390/bioengineering13101229)
Supplement: Supplementary file 1 [file bioengineering-13-01229-s001.zip › Supplementary_Material.pdf]

Article

Supplementary Materials: Nested Evaluation of Sparse Multi-Objective Evolutionary Gene-Panel Discovery for Diabetic Nephropathy

Yilei Cong [1,†], Tengfei Wu [1,†], Xinyu Yang [1], Yiyun Zhu [2], Min Tang [1], Hua Yang [1,*] and Ping Liu [3,*]

[1] Department of Endocrinology, Longhua Hospital, Shanghai University of Traditional Chinese Medicine, Shanghai 200032, China; congyl123@126.com (Y.C.); lh02182@shutcm.edu.cn (T.W.); melodyyang1993@163.com (X.Y.); tangmin_longhua@163.com (M.T.)

[2] Ward 2, Department of Nephrology, Longhua Hospital, Shanghai University of Traditional Chinese Medicine, Shanghai 200032, China; zyy9221@126.com

[3] Department of Cardiology, Longhua Hospital, Shanghai University of Traditional Chinese Medicine, Shanghai 200032, China

* Correspondence: yanghua@longhua.net (H.Y.); liuping0207@yeah.net (P.L.)

† These authors contributed equally to this work.

Abstract: This file supplements the within-run leakage-controlled three-objective sparse evolutionary analysis. It provides complete corrected algorithm comparisons, search-front comparisons, NSGA-II GO-objective and initialization ablations, feature recurrence, external feature-set portability summaries, and reproducibility details, together with the analyses added during revision: repeated search runs, comparator feature-selection methods, pathway over-representation, GO-aware representative rules, and robustness analyses.

Keywords: diabetic nephropathy; nested cross-validation; multi-objective optimization; sparse initialization; Gene Ontology

S1. Experimental matrix and retained outputs

All supervised discovery steps were repeated within each outer training partition of GSE96804. The formal experiment contained 160 optimization tasks: six algorithms under the real-GO three-objective formulation across 20 outer splits, plus no-GO and randomized-GO NSGA-II controls across the same splits. Each task retained its representative panel, complete final nondominated archive, configuration metadata, and split-level outer-test results. The main aggregate contained 5261 nondominated solutions. The initialization ablation added 60 newly executed control runs and reused the 20 completed stratified-harmonic NSGA-II real-GO runs. The revision added 960 runs: 480 additional search runs for the six algorithms, 180 runs on outer repeats five to ten, 100 permuted-label runs, 120 runs with inner-fold screening, 20 runs without the GSE30528 coverage restriction, and 60 runs for the bounded uniform and SparseEA initializers and the complete SparseEA algorithm. The five comparator feature-selection methods were fitted once per outer split.

Candidate-pool construction, harmonic sparse initialization, evolutionary variation, and representative selection followed the main manuscript. No cardinality cap or repair operator was used. Real-GO similarity was the mean Jaccard coefficient across all distinct gene pairs in a panel. Under random sampling its expectation equals the candidate-universe mean pairwise similarity and does not rise mechanically with panel size.

S2. Corrected algorithm comparisons

Table S1 gives every pairwise comparison for outer AUC, outer balanced accuracy, and representative size. Each comparison used the 20 matched outer splits and the Nadeau–Bengio corrected resampled t approximation. Holm adjustment was applied across the 15 algorithm pairs separately for each outcome.

Table S1. Complete corrected pairwise comparisons of representative-panel outcomes. Differences are first algorithm minus second algorithm.

Metric	First	Second	Mean difference	p_H
AUC	NSGA-II	NSGA-II tie-break	0.0014	1.0000
AUC	NSGA-II	SPEA2	0.0094	1.0000
AUC	NSGA-II	SPEA2+	-0.0016	1.0000
AUC	NSGA-II	MOEA/D	-0.0078	1.0000
AUC	NSGA-II	MOEA/D-NRD	-0.0042	1.0000
AUC	NSGA-II tie-break	SPEA2	0.0080	1.0000
AUC	NSGA-II tie-break	SPEA2+	-0.0030	1.0000
AUC	NSGA-II tie-break	MOEA/D	-0.0092	1.0000
AUC	NSGA-II tie-break	MOEA/D-NRD	-0.0056	1.0000
AUC	SPEA2	SPEA2+	-0.0109	1.0000
AUC	SPEA2	MOEA/D	-0.0172	1.0000
AUC	SPEA2	MOEA/D-NRD	-0.0135	1.0000
AUC	SPEA2+	MOEA/D	-0.0063	1.0000
AUC	SPEA2+	MOEA/D-NRD	-0.0026	1.0000
AUC	MOEA/D	MOEA/D-NRD	0.0036	1.0000
BA	NSGA-II	NSGA-II tie-break	-0.0128	1.0000
BA	NSGA-II	SPEA2	-0.0187	1.0000
BA	NSGA-II	SPEA2+	-0.0281	1.0000
BA	NSGA-II	MOEA/D	-0.0066	1.0000
BA	NSGA-II	MOEA/D-NRD	-0.0378	1.0000
BA	NSGA-II tie-break	SPEA2	-0.0059	1.0000
BA	NSGA-II tie-break	SPEA2+	-0.0153	1.0000
BA	NSGA-II tie-break	MOEA/D	0.0063	1.0000
BA	NSGA-II tie-break	MOEA/D-NRD	-0.0250	1.0000
BA	SPEA2	SPEA2+	-0.0094	1.0000
BA	SPEA2	MOEA/D	0.0122	1.0000
BA	SPEA2	MOEA/D-NRD	-0.0191	1.0000
BA	SPEA2+	MOEA/D	0.0215	1.0000
BA	SPEA2+	MOEA/D-NRD	-0.0097	1.0000
BA	MOEA/D	MOEA/D-NRD	-0.0312	1.0000
Size	NSGA-II	NSGA-II tie-break	-0.0500	1.0000
Size	NSGA-II	SPEA2	0.0000	1.0000
Size	NSGA-II	SPEA2+	0.0500	1.0000
Size	NSGA-II	MOEA/D	-0.4000	1.0000
Size	NSGA-II	MOEA/D-NRD	-0.5500	1.0000
Size	NSGA-II tie-break	SPEA2	0.0500	1.0000
Size	NSGA-II tie-break	SPEA2+	0.1000	1.0000
Size	NSGA-II tie-break	MOEA/D	-0.3500	1.0000
Size	NSGA-II tie-break	MOEA/D-NRD	-0.5000	1.0000
Size	SPEA2	SPEA2+	0.0500	1.0000
Size	SPEA2	MOEA/D	-0.4000	1.0000
Size	SPEA2	MOEA/D-NRD	-0.5500	1.0000
Size	SPEA2+	MOEA/D	-0.4500	1.0000
Size	SPEA2+	MOEA/D-NRD	-0.6000	1.0000
Size	MOEA/D	MOEA/D-NRD	-0.1500	1.0000

S3. Search-front comparisons

Table S2 reports the complete matched HV and empirical IGD contrasts. Objectives were normalized within each outer split using the pooled six-algorithm archive, so the indicators compare algorithms under a common split-specific scale.

Table S2. Complete corrected pairwise comparisons of three-objective front descriptors. Differences are first algorithm minus second algorithm.

Metric	First	Second	Mean difference	p_H
HV	NSGA-II	NSGA-II tie-break	-0.0264	1.0000
HV	NSGA-II	SPEA2	-0.0055	1.0000
HV	NSGA-II	SPEA2+	-0.0358	1.0000
HV	NSGA-II	MOEA/D	0.2278	0.1378
HV	NSGA-II	MOEA/D-NRD	0.3259	0.0374
HV	NSGA-II tie-break	SPEA2	0.0210	1.0000
HV	NSGA-II tie-break	SPEA2+	-0.0093	1.0000
HV	NSGA-II tie-break	MOEA/D	0.2542	0.1226
HV	NSGA-II tie-break	MOEA/D-NRD	0.3523	0.0212
HV	SPEA2	SPEA2+	-0.0303	1.0000
HV	SPEA2	MOEA/D	0.2333	0.0697
HV	SPEA2	MOEA/D-NRD	0.3313	0.0056
HV	SPEA2+	MOEA/D	0.2635	0.1063
HV	SPEA2+	MOEA/D-NRD	0.3616	0.0118
HV	MOEA/D	MOEA/D-NRD	0.0981	1.0000
IGD	NSGA-II	NSGA-II tie-break	0.0138	1.0000

Metric	First	Second	Mean difference	p_H
IGD	NSGA-II	SPEA2	-0.0036	1.0000
IGD	NSGA-II	SPEA2+	0.0218	1.0000
IGD	NSGA-II	MOEA/D	-0.1939	0.2281
IGD	NSGA-II	MOEA/D-NRD	-0.2561	0.1376
IGD	NSGA-II tie-break	SPEA2	-0.0174	1.0000
IGD	NSGA-II tie-break	SPEA2+	0.0080	1.0000
IGD	NSGA-II tie-break	MOEA/D	-0.2077	0.1849
IGD	NSGA-II tie-break	MOEA/D-NRD	-0.2698	0.0783
IGD	SPEA2	SPEA2+	0.0254	1.0000
IGD	SPEA2	MOEA/D	-0.1903	0.2281
IGD	SPEA2	MOEA/D-NRD	-0.2524	0.1089
IGD	SPEA2+	MOEA/D	-0.2157	0.2281
IGD	SPEA2+	MOEA/D-NRD	-0.2778	0.2281
IGD	MOEA/D	MOEA/D-NRD	-0.0621	1.0000

S4. GO-objective ablation

Table S3 reports representative-panel and archive-level NSGA-II contrasts. All panels were rescored with unpermuted real-GO annotation. Archive maximum, mean, and 90th percentile quantify target attainment across the nondominated front.

Table S3. Corrected NSGA-II GO-ablation contrasts. Differences are real GO minus the stated control.

Level/metric	Contrast	Mean difference	95% CI low	95% CI high	p_H
Representative AUC	go_real - no_go	-0.0047	-0.0486	0.0392	1.0000
Representative AUC	go_real - go_randomized	-0.0111	-0.0532	0.0310	1.0000
Representative BA	go_real - no_go	-0.0281	-0.1281	0.0718	1.0000
Representative BA	go_real - go_randomized	-0.0031	-0.1336	0.1273	1.0000
Representative Size	go_real - no_go	0.0500	-0.5352	0.6352	1.0000
Representative Size	go_real - go_randomized	0.1000	-0.4128	0.6128	1.0000
Representative GO	go_real - no_go	0.0890	-0.0614	0.2394	0.4614
Representative GO	go_real - go_randomized	0.0776	-0.0878	0.2430	0.4614
Archive maximum GO	go_real - no_go	0.9159	0.7960	1.0359	3.57e-12
Archive maximum GO	go_real - go_randomized	0.9071	0.7827	1.0315	4.06e-12
Archive mean GO	go_real - no_go	0.4452	0.3216	0.5687	7.98e-07
Archive mean GO	go_real - go_randomized	0.4476	0.3198	0.5754	7.98e-07
Archive 90th percentile GO	go_real - no_go	0.7440	0.6021	0.8859	2.30e-09
Archive 90th percentile GO	go_real - go_randomized	0.7464	0.6012	0.8916	2.30e-09

S5. Feature recurrence

Table S4 lists genes appearing in at least two of an algorithm's 20 real-GO representative panels. No gene reached 50% recurrence. Counts describe stability across overlapping outer training partitions.

Table S4. Genes recurring in at least two real-GO representative panels for an algorithm.

Algorithm	Gene	Splits	Frequency
MOEA/D	DUSP1	4/20	0.20
MOEA/D	CPPED1	2/20	0.10
MOEA/D	FOS	2/20	0.10
MOEA/D	NDUFAB1	2/20	0.10
MOEA/D-NRD	DUSP1	6/20	0.30
MOEA/D-NRD	FOS	3/20	0.15
MOEA/D-NRD	C11orf1	2/20	0.10
MOEA/D-NRD	NFIC	2/20	0.10
MOEA/D-NRD	PAXBP1	2/20	0.10
MOEA/D-NRD	PTOV1	2/20	0.10
MOEA/D-NRD	SALL1	2/20	0.10
NSGA-II	FOS	4/20	0.20
NSGA-II	NFATC3	4/20	0.20
NSGA-II	CNBP	3/20	0.15
NSGA-II	CHCHD2	2/20	0.10
NSGA-II	CPPED1	2/20	0.10
NSGA-II	DUSP1	2/20	0.10
NSGA-II	PTOV1	2/20	0.10
NSGA-II	S100A8	2/20	0.10
NSGA-II tie-break	CNBP	4/20	0.20
NSGA-II tie-break	DUSP1	4/20	0.20
NSGA-II tie-break	FOS	4/20	0.20
NSGA-II tie-break	CHCHD2	2/20	0.10
NSGA-II tie-break	PTOV1	2/20	0.10

Algorithm	Gene	Splits	Frequency
SPEA2	DUSP1	6/20	0.30
SPEA2	NFATC3	4/20	0.20
SPEA2	CNBP	3/20	0.15
SPEA2	FOS	3/20	0.15
SPEA2	EED	2/20	0.10
SPEA2	JUND	2/20	0.10
SPEA2	PEBP1	2/20	0.10
SPEA2+	DGKH	3/20	0.15
SPEA2+	NFATC3	3/20	0.15
SPEA2+	CHCHD2	2/20	0.10
SPEA2+	DUSP1	2/20	0.10
SPEA2+	FOS	2/20	0.10
SPEA2+	PDK4	2/20	0.10
SPEA2+	PEBP1	2/20	0.10
SPEA2+	PTOV1	2/20	0.10

S6. External feature-set portability

Table S5 summarizes cohort-specific five-fold refitting in GSE30528 and GSE142025. Gene identities were fixed before external labels were used, while scalers and classifier coefficients were estimated within each external fold. Locked-model transfer is reported in the main text, and all panel-level external results, including sensitivity, specificity, and prevalence-threshold balanced accuracy, are provided in Supplementary Data file S9. In file S9, the top-DEG rows consist of the first $|S|$ discovery-ranked genes, from which genes absent from the external cohort were removed; the top-DEG controls in main-text Table 10 were instead drawn from the available genes at the effective panel size, which differs in a few GSE142025 splits.

Table S5. External cohort-specific refitting summaries for the real-GO representative panels.

Cohort	Algorithm	Effective size	AUC	BA	ΔAUC random
GSE142025	MOEA/D	2.45	0.868	0.618	0.032
GSE142025	MOEA/D-NRD	2.65	0.964	0.643	0.109
GSE142025	NSGA-II	2.15	0.880	0.580	0.048
GSE142025	NSGA-II tie-break	2.20	0.914	0.606	0.080
GSE142025	SPEA2	2.15	0.881	0.616	0.049
GSE142025	SPEA2+	2.05	0.849	0.572	0.024
GSE30528	MOEA/D	2.55	0.726	0.658	-0.005
GSE30528	MOEA/D-NRD	2.70	0.694	0.625	-0.048
GSE30528	NSGA-II	2.15	0.726	0.628	0.027
GSE30528	NSGA-II tie-break	2.20	0.657	0.598	-0.044
GSE30528	SPEA2	2.15	0.650	0.621	-0.050
GSE30528	SPEA2+	2.10	0.612	0.582	-0.086

S7. Initialization ablation

Table S6 gives the complete matched final-budget contrasts. The controls were IID harmonic sampling, expected-size-matched Bernoulli sampling with $p = 1/H_m$, and standard dense Bernoulli sampling with $p = 0.5$. The stratified-harmonic rows were reused from the completed main experiment; only the 60 controls were newly executed. Front indicators were recalculated on a common four-initializer, split-specific normalized scale. The main text reports the extended seven-condition comparison, which adds the bounded uniform initializer, the SparseEA initializer, and the complete SparseEA algorithm, with joint normalization across all seven conditions; its split-level values are in Supplementary Data file S6. Because the four-condition normalization differs, the HV and IGD values in Table S6 differ from those in the main text and in file S6, while sizes and outer metrics are identical. The p_{H} values also differ, because Holm adjustment here spans three controls instead of six and the original correction factor of 0.3001 was used.

Table S6. Corrected NSGA-II initialization-ablation contrasts. Differences are stratified harmonic minus the stated control. Holm adjustment was applied across the three controls separately for each metric.

Control	Metric	Harmonic mean	Control mean	Difference (95% corrected CI)	p_{H}
IID harmonic	HV	0.7213	0.7739	−0.0526 [−0.2434, 0.1382]	0.571
IID harmonic	IGD	0.0890	0.0458	0.0432 [−0.0834, 0.1698]	0.484
IID harmonic	Outer AUC	0.9828	0.9799	0.0030 [−0.0583, 0.0643]	0.982
IID harmonic	Outer BA	0.8938	0.9128	−0.0191 [−0.1638, 0.1256]	0.785
IID harmonic	Size	2.1500	2.1500	0.0000 [−0.6443, 0.6443]	1.000
Mean-matched Bernoulli	HV	0.7213	0.0287	0.6926 [0.5819, 0.8033]	1.25×10^{-10}
Mean-matched Bernoulli	IGD	0.0890	0.8024	−0.7134 [−0.8162, −0.6105]	1.95×10^{-11}
Mean-matched Bernoulli	Outer AUC	0.9828	1.0000	−0.0172 [−0.0530, 0.0186]	0.982
Mean-matched Bernoulli	Outer BA	0.8938	0.9972	−0.1035 [−0.2125, 0.0055]	0.138
Mean-matched Bernoulli	Size	2.1500	38.0000	−35.8500 [−46.1272, −25.5728]	1.27×10^{-6}
Dense Bernoulli	HV	0.7213	0.0121	0.7092 [0.5981, 0.8204]	1.25×10^{-10}
Dense Bernoulli	IGD	0.0890	0.8697	−0.7807 [−0.8872, −0.6743]	1.10×10^{-11}
Dense Bernoulli	Outer AUC	0.9828	1.0000	−0.0172 [−0.0530, 0.0186]	0.982
Dense Bernoulli	Outer BA	0.8938	1.0000	−0.1063 [−0.2105, −0.0020]	0.138
Dense Bernoulli	Size	2.1500	360.7000	−358.5500 [−405.1613, −311.9387]	4.72×10^{-12}

S8. Reproducibility details

The outer resampling seed was 20260920. Evolutionary searches used seed $20261000 + s$, same-size random-panel controls used a deterministic sequence beginning at 20262000, and randomized GO used seed $20263000 + s$, where s is the outer-split index. Each search used a 10,000-evaluation target, population size 100 for the NSGA-II and SPEA2 families, 105 reference directions for the three-objective MOEA/D family, two-point crossover probability 0.9, and bit-flip mutation probability $1/m$.

The formal aggregate comprised 120 real-GO main rows and 40 NSGA-II GO-ablation rows. The separate initialization ablation comprised 60 new control rows paired with the 20 existing stratified-harmonic NSGA-II real-GO rows. Validation required one representative file, one Pareto archive, and one metadata file for every task index before aggregation.

Additional search runs used seed $20261000 + s + 1000r$ for run $r = 1, \ldots, 4$, with the inner-CV partition fixed at the primary seed $20261000 + s$. Outer repeats five to ten came from the same RepeatedStratifiedKFold object with random state 20260920 and ten repeats, which reproduces the first 20 splits exactly. Permuted labels used seed $20264000 + k$ for permutation k, and random-panel expectations for annotation coherence used seed 20265000. Comparator methods used seed $20261000 + s$ for their inner folds and subsamples.

All analyses ran with Python 3.14.6, NumPy 2.5.3, SciPy 1.18.1, pandas 3.0.6, scikit-learn 1.9.1, pymoo 0.6.2, statsmodels 0.15.0, GEOparse 2.0.4, and Matplotlib 3.11.2. Processed series matrices were downloaded from GEO with GEOparse; probe identifiers were mapped to gene symbols from the GPL17586 gene assignment field and the GPL571 gene symbol column, and multiple probes per symbol were averaged. The GSE142025 matrix was taken from the series supplementary file, and its labels were checked against the series metadata. Gene-set libraries were Enrichr files, listed below with their MD5 checksums. Split-level results of every analysis are provided in the file Supplementary_Data.zip, files S1 to S12.

- GO_Biological_Process_2023: `1d0c46f1bbf5b371001ccf19dc4bb3e7`
- Reactome_2022: `faeaf930a151f179ee18e4efc6ce3866`
- KEGG_2021_Human: `c3551d68425f44f23284d36d7f26d7f1`
- MSigDB_Hallmark_2020: `a07e950680738a6ce60aedac9b96f8c9`

S9. Repeated search runs

Table S7 summarizes the five independent runs per algorithm and outer split. Variation between runs on the same split was of the same size as variation between splits. Front indicators were recomputed with joint normalization across the 30 runs of each split.

Table S7. Five independent search runs per algorithm and outer split. AUC and BA are means over 100 runs; the range gives the smallest and largest mean over the five run indices. Within-split SD is the standard deviation across the five runs on one split, averaged over splits. HV and IGD use joint normalization over all 30 runs of a split.

Algorithm	AUC	Run-mean range	BA	BA SD within	Size	Size SD within	HV	HV SD within	IGD
NSGA-II	0.986	0.981–0.989	0.909	0.078	2.18	0.24	0.678	0.068	0.140
NSGA-II tie-break	0.985	0.980–0.991	0.918	0.079	2.18	0.24	0.691	0.066	0.126
SPEA2	0.983	0.968–1.000	0.919	0.060	2.18	0.25	0.691	0.064	0.128
SPEA2+	0.987	0.975–0.994	0.921	0.075	2.11	0.17	0.700	0.064	0.123
MOEA/D	0.987	0.977–0.994	0.901	0.093	2.84	0.70	0.467	0.117	0.313
MOEA/D-NRD	0.988	0.983–0.991	0.901	0.095	2.53	0.52	0.357	0.125	0.396

S10. Comparator feature-selection methods

Table S8 gives the paired contrasts between each evolutionary algorithm and each comparator method on the 20 outer splits. Comparator gene sets were scored with the same standardized L2 logistic-regression classifier with $C = 0.1$.

Table S8. Paired differences between evolutionary panels and established feature-selection methods on the same 20 outer splits. Each cell gives the mean difference, evolutionary minus baseline, and the Holm-adjusted p_{H} from the Nadeau–Bengio corrected resampled t approximation, adjusted across six algorithms for each baseline and metric. Selected penalties: LASSO C median 0.070; elastic net C median 0.046, mixing parameter median 0.1.

Baseline	Metric	NSGA-II	NSGA-II tie-break	SPEA2	SPEA2+	MOEA/D	MOEA/D-NRD
LASSO	AUC	−0.017; 1.000	−0.019; 1.000	−0.027; 1.000	−0.016; 1.000	−0.009; 1.000	−0.013; 1.000
	BA	−0.056; 1.000	−0.043; 1.000	−0.037; 1.000	−0.028; 1.000	−0.050; 1.000	−0.018; 1.000
Elastic net	AUC	−0.017; 1.000	−0.019; 1.000	−0.027; 1.000	−0.016; 1.000	−0.009; 1.000	−0.013; 1.000
	BA	−0.106; 0.2767	−0.093; 0.3292	−0.087; 0.4503	−0.078; 0.4503	−0.100; 0.4503	−0.068; 0.4503
RFE, matched size	AUC	−0.016; 1.000	−0.017; 1.000	−0.025; 1.000	−0.014; 1.000	−0.008; 1.000	−0.011; 1.000
	BA	0.025; 1.000	0.038; 1.000	0.044; 1.000	0.053; 1.000	0.032; 1.000	0.063; 1.000
RFECV	AUC	−0.017; 1.000	−0.019; 1.000	−0.027; 1.000	−0.016; 1.000	−0.009; 1.000	−0.013; 1.000
	BA	−0.106; 0.2767	−0.093; 0.3292	−0.087; 0.4503	−0.078; 0.4503	−0.100; 0.4503	−0.068; 0.4503
Stability selection	AUC	−0.017; 1.000	−0.019; 1.000	−0.027; 1.000	−0.016; 1.000	−0.009; 1.000	−0.013; 1.000
	BA	0.056; 1.000	0.069; 1.000	0.075; 1.000	0.084; 1.000	0.063; 1.000	0.094; 0.9121

S11. Pathway over-representation of selected genes

Table S9 lists the over-represented terms for genes selected under real GO, without GO, and with randomized GO, and for genes selected in at least three of the 600 runs of the five-run analysis. The background was the union of the 20 candidate pools. Because candidate pools contain only differentially expressed genes, these tests ask which programs the search favored among differentially expressed genes.

Table S9. Over-representation of selected genes in Reactome 2022, KEGG 2021 Human, and MSigDB Hallmark 2020 against the union of the 20 candidate pools, 1686 genes. Overlap gives selected genes in the term over pool genes in the term; FDR is the Benjamini–Hochberg adjustment within each library and query. Terms with FDR < 0.05 are shown.

Query	Library	Term	Overlap	FDR	Genes
Real-GO panels, six algorithms ($n = 121$)	Reactome	Complex I Biogenesis	6/8	0.0018	NDUFA2, NDUFAB1, NDUFB5, NDUFB7, NDUFC1, NDUFV1
	Reactome	ESR-mediated Signaling	8/16	0.0018	CBFB, EGF, FKBP4, FOS, FOSB, JUN, JUND, PIK3R3
	KEGG	Non-alcoholic fatty liver disease	11/33	0.0024	FOS, JUN, NDUFA2, NDUFAB1, NDUFB5, NDUFB7, NDUFC1, NDUFV1, PIK3R3, SDHC, UQCR10
	Reactome	Estrogen-dependent Gene Expression	6/10	0.0055	CBFB, FKBP4, FOS, FOSB, JUN, JUND
	Reactome	Signaling By Nuclear Receptors	10/32	0.0087	CBFB, EGF, FKBP4, FOS, FOSB, JUN, JUND, PDK2, PDK4, PIK3R3
	KEGG	Diabetic cardiomyopathy	11/46	0.0253	NDUFA2, NDUFAB1, NDUFB5, NDUFB7, NDUFC1, NDUFV1, PDK2, PDK4, PIK3R3, SDHC, UQCR10
	KEGG	Oxidative phosphorylation	8/26	0.0253	NDUFA2, NDUFAB1, NDUFB5, NDUFB7, NDUFC1, NDUFV1, SDHC, UQCR10
	KEGG	Retrograde endocannabinoid signaling	6/15	0.0253	NDUFA2, NDUFAB1, NDUFB5, NDUFB7, NDUFC1, NDUFV1
	KEGG	Amyotrophic lateral sclerosis	10/42	0.0272	ANG, GABARAP, NDUFA2, NDUFAB1, NDUFB5, NDUFB7, NDUFC1, NDUFV1, SDHC, UQCR10
	KEGG	PD-L1 expression and PD-1 checkpoint pathway in cancer	5/11	0.0272	EGF, FOS, JUN, NFATC3, PIK3R3
	KEGG	Amphetamine addiction	4/7	0.0273	FOS, FOSB, JUN, MAOA
	KEGG	Parkinson disease	10/44	0.0273	DUSP1, MAOA, NDUFA2, NDUFAB1, NDUFB5, NDUFB7, NDUFC1, NDUFV1, SDHC, UQCR10
	Hallmark	TNF-alpha Signaling via NF-kB	12/59	0.0333	ATF3, BTG2, DUSP1, FOS, FOSB, HES1, INHBA, JUN, MXD1, NFIL3, PNRC1, ZFP36
	KEGG	Cocaine addiction	3/4	0.0405	FOSB, JUN, MAOA
	KEGG	Choline metabolism in cancer	5/13	0.0405	DGKH, EGF, FOS, JUN, PIK3R3
	KEGG	Thermogenesis	8/33	0.0439	NDUFA2, NDUFAB1, NDUFB5, NDUFB7, NDUFC1, NDUFV1, SDHC, UQCR10

Query	Library	Term	Overlap	FDR	Genes
	KEGG	Prion disease	10/49	0.0443	HSPA1L, NDUFA2, NDUFAB1, NDUFB5, NDUFB7, NDUFC1, NDUFV1, PIK3R3, SDHC, UQCR10
	KEGG	Estrogen signaling pathway	5/14	0.0457	FKBP4, FOS, HSPA1L, JUN, PIK3R3
	KEGG	B cell receptor signaling pathway	4/9	0.0457	FOS, JUN, NFATC3, PIK3R3
	KEGG	Signaling pathways regulating pluripotency of stem cells	4/9	0.0457	FGFR4, ID4, INHBA, PIK3R3
No-GO NSGA-II panels ($n = 32$)	Hallmark	Oxidative Phosphorylation	6/63	0.0453	COX6B1, ECHS1, MRPL11, NDUFAB1, NDUFC1, UQCR10
Randomized-GO NSGA-II panels	–	No term with FDR < 0.05			
Genes in at least 3 of 600 runs ($n = 105$)	KEGG	Non-alcoholic fatty liver disease	11/33	0.0006	FOS, JUN, NDUFA7, NDUFAB1, NDUFB2, NDUFB5, NDUFB7, NDUFC1, SDHB, SDHC, UQCR10
	KEGG	Amyotrophic lateral sclerosis	12/42	0.0006	GABARAP, GABARAPL1, NDUFA7, NDUFAB1, NDUFB2, NDUFB5, NDUFB7, NDUFC1, SDHB, SDHC, SOD1, UQCR10
	Hallmark	Oxidative Phosphorylation	14/63	0.0008	ECH1, ECI1, MRPL11, MRPL34, NDUFA7, NDUFAB1, NDUFB2, NDUFB5, NDUFB7, NDUFC1, PDK4, SDHB, SDHC, UQCR10
	Reactome	Complex I Biogenesis	6/8	0.0011	NDUFA7, NDUFAB1, NDUFB2, NDUFB5, NDUFB7, NDUFC1
	KEGG	Oxidative phosphorylation	9/26	0.0012	NDUFA7, NDUFAB1, NDUFB2, NDUFB5, NDUFB7, NDUFC1, SDHB, SDHC, UQCR10
	KEGG	Diabetic cardiomyopathy	11/46	0.0045	NDUFA7, NDUFAB1, NDUFB2, NDUFB5, NDUFB7, NDUFC1, PDK2, PDK4, SDHB, SDHC, UQCR10
	KEGG	Huntington disease	10/39	0.0045	NDUFA7, NDUFAB1, NDUFB2, NDUFB5, NDUFB7, NDUFC1, SDHB, SDHC, SOD1, UQCR10
	KEGG	Thermogenesis	9/33	0.0052	NDUFA7, NDUFAB1, NDUFB2, NDUFB5, NDUFB7, NDUFC1, SDHB, SDHC, UQCR10
	KEGG	Retrograde endocannabinoid signaling	6/15	0.0065	NDUFA7, NDUFAB1, NDUFB2, NDUFB5, NDUFB7, NDUFC1
	KEGG	Parkinson disease	10/44	0.0085	DUSP1, NDUFA7, NDUFAB1, NDUFB2, NDUFB5, NDUFB7, NDUFC1, SDHB, SDHC, UQCR10
	KEGG	Pathways of neurodegeneration	12/62	0.0090	GABARAP, GABARAPL1, NDUFA7, NDUFAB1, NDUFB2, NDUFB5, NDUFB7, NDUFC1, SDHB, SDHC, SOD1, UQCR10
	Reactome	Respiratory Electron Transport	8/24	0.0153	NDUFA7, NDUFAB1, NDUFB2, NDUFB5, NDUFB7, NDUFC1, SDHB, UQCR10
	Reactome	Respiratory Electron Transport, ATP Synthesis By Chemiosmotic Coupling, Heat Production By Uncoupling Proteins	8/24	0.0153	NDUFA7, NDUFAB1, NDUFB2, NDUFB5, NDUFB7, NDUFC1, SDHB, UQCR10
	Reactome	Citric Acid (TCA) Cycle And Respiratory Electron Transport	10/39	0.0164	NDUFA7, NDUFAB1, NDUFB2, NDUFB5, NDUFB7, NDUFC1, PDK2, PDK4, SDHB, UQCR10
	KEGG	Prion disease	10/49	0.0173	NDUFA7, NDUFAB1, NDUFB2, NDUFB5, NDUFB7, NDUFC1, SDHB, SDHC, SOD1, UQCR10
	Hallmark	TNF-alpha Signaling via NF-kB	11/59	0.0181	ATF3, BTG2, DUSP1, FOS, FOSB, JUN, JUNB, MXD1, NFIL3, PNRC1, ZFP36
	Reactome	Estrogen-dependent Nuclear Events Downstream Of ESR-membrane Signaling	3/3	0.0335	CDKN1B, EGF, FOS
	Reactome	ESR-mediated Signaling	6/16	0.0335	CDKN1B, EGF, FOS, FOSB, JUN, JUND
	KEGG	IL-17 signaling pathway	5/15	0.0403	FOS, FOSB, JUN, JUND, S100A8

S12. GO-aware representative rules and independent annotation

Table S10 applies the accuracy-first rule, the tolerance rule with $\delta = 0.025$ and $\delta = 0.05$, and the archive-maximum GO panel to every final archive, and reports coherence under Reactome, KEGG, and Hallmark annotations.

Table S10. Representative rules applied to the final archives and coherence under annotation resources not used during search. Values are means over 20 outer splits. p_{H} refers to the change in real-GO coherence relative to the accuracy-first panel, Holm-adjusted across algorithms; the last rows compare the archive maximum of each independent coherence between NSGA-II conditions.

Archive	Rule	Size	Real GO	p_{H}	AUC	BA	Reactome	KEGG	Hallmark
Randomized GO, NSGA-II	Accuracy first	2.05	0.020	–	0.994	0.897	0.008	0.000	0.000
	Tolerance $\delta = 0.025$	3.05	0.032	0.1888	0.995	0.903	0.003	0.001	0.010
	Tolerance $\delta = 0.05$	3.00	0.032	0.1761	0.995	0.903	0.003	0.001	0.009
	Archive maximum GO	2.60	0.053	–	0.977	0.844	0.007	0.001	0.079
Real GO, MOEA/D	Accuracy first	2.55	0.094	–	0.991	0.900	0.053	0.049	0.021
	Tolerance $\delta = 0.025$	4.40	0.288	0.0496	0.988	0.922	0.151	0.151	0.080
	Tolerance $\delta = 0.05$	4.15	0.357	0.0097	0.991	0.910	0.189	0.191	0.092
	Archive maximum GO	2.55	0.650	–	0.963	0.848	0.232	0.278	0.107
Real GO, MOEA/D-NRD	Accuracy first	2.70	0.156	–	0.987	0.932	0.025	0.005	0.010
	Tolerance $\delta = 0.025$	3.20	0.198	0.2203	0.989	0.944	0.042	0.015	0.013
	Tolerance $\delta = 0.05$	3.15	0.226	0.1806	0.984	0.919	0.051	0.038	0.012
	Archive maximum GO	2.40	0.480	–	0.970	0.869	0.095	0.054	0.017
Real GO, NSGA-II	Accuracy first	2.15	0.098	–	0.983	0.894	0.016	0.019	0.017
	Tolerance $\delta = 0.025$	4.40	0.620	0.0006	0.975	0.884	0.072	0.067	0.057
	Tolerance $\delta = 0.05$	4.10	0.668	0.0003	0.975	0.856	0.076	0.058	0.052
	Archive maximum GO	2.05	0.960	–	0.908	0.695	0.039	0.100	0.128
Real GO, NSGA-II tie-break	Accuracy first	2.20	0.119	–	0.981	0.907	0.004	0.017	0.017
	Tolerance $\delta = 0.025$	4.30	0.618	0.0006	0.980	0.894	0.070	0.091	0.069
	Tolerance $\delta = 0.05$	3.75	0.698	0.0006	0.961	0.897	0.062	0.102	0.079
	Archive maximum GO	2.05	0.954	–	0.922	0.835	0.111	0.061	0.113
Real GO, SPEA2	Accuracy first	2.15	0.119	–	0.973	0.912	0.000	0.002	0.000
	Tolerance $\delta = 0.025$	4.75	0.592	0.0006	0.989	0.934	0.062	0.092	0.064
	Tolerance $\delta = 0.05$	4.30	0.655	0.0001	0.981	0.847	0.067	0.102	0.081
	Archive maximum GO	2.05	0.958	–	0.916	0.698	0.097	0.100	0.126
Real GO, SPEA2+	Accuracy first	2.10	0.127	–	0.984	0.922	0.023	0.020	0.017
	Tolerance $\delta = 0.025$	4.50	0.636	0.0006	0.975	0.916	0.141	0.176	0.143
	Tolerance $\delta = 0.05$	4.05	0.662	0.0003	0.966	0.869	0.133	0.177	0.143
	Archive maximum GO	2.10	0.966	–	0.950	0.819	0.208	0.150	0.152
No GO, NSGA-II	Accuracy first	2.10	0.009	–	0.988	0.922	0.008	0.001	0.050
	Tolerance $\delta = 0.025$	2.10	0.044	0.2919	0.988	0.941	0.011	0.003	0.050
	Tolerance $\delta = 0.05$	2.10	0.044	0.2919	0.988	0.941	0.011	0.003	0.050
	Archive maximum GO	1.80	0.044	–	0.958	0.866	0.003	0.003	0.000
Archive maximum Reactome, NSGA-II, real vs no GO			0.198 vs 0.036	0.3903					
Archive maximum Reactome, NSGA-II, real vs randomized GO			0.198 vs 0.058	0.3903					
Archive maximum KEGG, NSGA-II, real vs no GO			0.306 vs 0.029	0.5140					
Archive maximum KEGG, NSGA-II, real vs randomized GO			0.306 vs 0.022	0.5140					
Archive maximum Hallmark, NSGA-II, real vs no GO			0.274 vs 0.106	1.000					
Archive maximum Hallmark, NSGA-II, real vs randomized GO			0.274 vs 0.122	1.000					

S13. Robustness of internal estimates

Figure S1 shows the cumulative outer-fold results as repeats five to ten were added and the distribution of outer AUC under permuted labels.

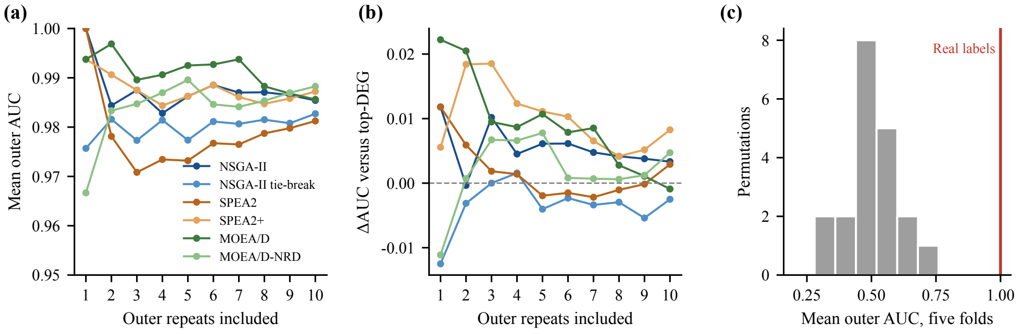

Figure S1. Robustness of internal estimates. (**a**) Mean outer AUC of each algorithm as outer repeats are added, from 5 to 50 splits. (**b**) Mean AUC difference from the same-size top-DEG panel. (**c**) Mean outer AUC of NSGA-II over the five folds of the first repeat for 20 label permutations, with the value for the real labels marked in red.
